# Early-Life Hygiene-Related Factors and Risk of Inflammatory Bowel Disease: A Scandinavian Birth Cohort Study

**DOI:** 10.1093/ibd/izad257

**Published:** 2023-10-31

**Authors:** Annie Guo, Malin Östensson, Ketil Størdal, Johnny Ludvigsson, Karl Mårild

**Affiliations:** Department of Pediatrics, Institute of Clinical Sciences, Sahlgrenska Academy, University of Gothenburg, Gothenburg, Sweden; Bioinformatics and Data Centre, Sahlgrenska Academy, University of Gothenburg, Gothenburg, Sweden; Department of Pediatric Research, Faculty of Medicine, University of Oslo, Oslo, Norway; Children’s Center, Oslo University Hospital, Oslo, Norway; Crown Princess Victoria Children’s Hospital and Div of Pediatrics, Department of Biomedical and Clinical Sciences, Linköping University, Linköping, Sweden; Division of Pediatrics, Department of Biomedical and Clinical Sciences, Linköping University, Linköping, Sweden; Department of Pediatrics, Institute of Clinical Sciences, Sahlgrenska Academy, University of Gothenburg, Gothenburg, Sweden; Department of Pediatrics, Queen Silvia Children’s Hospital, Gothenburg, Sweden

**Keywords:** Inflammatory bowel disease, hygiene factors, epidemiology, early life

## Abstract

**Background:**

We aimed to investigate whether early-life hygiene-related factors influenced the risk of inflammatory bowel disease (IBD) in a Scandinavian population and test the association’s consistency across cohorts.

**Methods:**

This study followed 117 493 participants in the All Babies in Southeast Sweden study and the Norwegian Mother, Father, and Child Cohort Study. IBD diagnoses were defined by national registers. Comprehensive data on hygiene-related exposures, such as having pets, rural living, daycare attendance, and siblings, were retrieved from questionnaires administered from pregnancy until child’s age of 36 months. A multivariable Cox regression model yielded adjusted hazard ratios (aHRs) for IBD accounting for socioeconomic status and perinatal factors. Cohort-specific estimates were pooled using a random-effects model.

**Results:**

In over 2 024 299 person-years of follow-up 451 participants developed IBD. In pooled estimates children attending daycare up to 36 months of life vs not attending daycare were less likely to develop Crohn’s disease (aHR, 0.60; 95% confidence interval [CI], 0.37- 0.98). Children having 1 or more siblings had a modestly increased risk of IBD (aHR, 1.17; 95% CI, 0.96-1.42; aHR for each sibling, 1.12; 95% CI, 1.01-1.24). The other hygiene factors were not significantly linked to later IBD. In the Norwegian Mother, Father, and Child Cohort Study cohort, bed sharing was associated with an increased risk of IBD, most notably for ulcerative colitis (aHR, 1.67; 95% CI, 1.01-2.78).

**Conclusions:**

In this birth cohort study from 2 high-income Scandinavian countries, some early-life hygiene-related exposures were associated with IBD risk. The generalizability of these results to countries of other socioeconomic level is unknown.

**Key Messages**

**What is already known?**

The increasing incidence of inflammatory bowel disease (IBD) suggests an influence of environmental exposures on disease development.

**What is new here?**

This Scandinavian cohort study following >117 000 participants from birth throughout childhood observed that having an older sibling was associated with an increased risk of IBD, while children who start daycare before they turn 36 months have a reduced risk of later IBD compared with children without daycare attendance.

**How can this study help patient care?**

Identification of risk factors is important for the future prevention of IBD.

## Introduction

Inflammatory bowel disease (IBD) is a chronic, relapsing inflammatory disorder comprising mainly 2 related subgroups: Crohn’s disease (CD) and ulcerative colitis (UC). Over the past decades, the incidence of IBD has increased^1^ and estimated to affect 0.3% of Western populations and is an emerging global disease. While genetic susceptibility plays a part in IBD,^2^ this rapid rise of IBD suggests an important role of environmental factors.^3^ The hygiene hypothesis, commonly attributed to Strachan, suggests that diminished microbial exposure in early life increases the risk of immune-mediated disease, including IBD.^4^

Early life is critical for adequate microbial colonization and imprinting of the developing immune system, which has been implicated in the susceptibility to IBD later in life.^5,6^ While the rising IBD incidence over the past decades has paralleled our changing lifestyle,^5,7^ individual-level data linking hygiene-related factors, including urban vs rural living and early-life pet exposure, with later IBD have been inconsistent (previous studies on children are summarized in Supplementary Table 1). There are also possibilities that some other factors are directly or indirectly linked to hygiene (eg, exposure to antibiotics, infections, parental education level, and breastfeeding status).^8^

Previous data have been mainly based on retrospective designs liable to recall and selection bias and included few population-based estimates. Casual inferences with observational data are also often hampered by confounding. Using population-based birth cohort data from 2 Scandinavian high-income countries, we investigated whether early-life country-specific hygiene-related factors were associated with subsequent IBD risk and tested the association’s consistency across 2 cohorts.

## Methods

### Study Population

We took advantage of prospectively collected data from the All Babies in Southeast Sweden (ABIS) study and the Norwegian Mother, Father and Child Cohort Study (MoBa) to examine the association of early-life hygiene-related factors with the risk of IBD (Supplementary Figure 1). The ABIS study is a population-based birth cohort in which all parents of children born between October 1, 1997, and October 1, 1999, in Southeast Sweden were asked to participate. Of some 21 700 children born during the 2-year study period, 17 055 (78.6%) were included after parental informed consent was obtained.^9^ Data from questionnaires administered at birth and when the child was 12 and 30 to 36 months of age were analyzed. We also linked data in the ABIS study to the Swedish National Patient Register^10^ and the Swedish Medical Birth Register.^11^ MoBa is a nationwide birth cohort administered by the Norwegian Institute of Public Health comprising 114 500 children born from 1999 to 2009 (participation rate 41%).^12^ This study used data from questionnaires administered in MoBa at weeks 15, 22, and 30 of pregnancy and when the child was 6, 18, and 36 months of age. MoBa was also linked to the Norwegian Patient Registry^13^ and the Medical Birth Registry of Norway (MBRN).^14^ Informed consent was retrieved from guardians of all ABIS and MoBa participants after written and oral information.

### Exposures

For the ABIS study, data on hygiene-related exposures were collected through questionnaires administered at birth and when the child was 12 and 30 to 36 months of age. In MoBa, the same data were collected through questionnaires administered at the 15th week of pregnancy; when the child was 6, 18, and 36 months of age; and from the MBRN (Supplementary Table 2).^14^ Motivated by previous research,^5^ we examined the following hygiene-related factors: daycare attendance (vs no attendance) by age 12 and 36 months; having 1 or more siblings at birth (vs no siblings), sibling order at birth (0, 1, 2, ≥3 older siblings), and per number of older siblings; having pets at birth (any pet vs no pet), the type of pets at birth (cat, dog, other/multiple vs no pet), and the duration of pet exposure by 36 months of age (≥2 years, 1 year vs 0 years [no pet]); early-life household crowding, defined as <25 m^2^ living area per person based on Norwegian standards,^15^ vs 25-50 m^2^ per person and >50 m^2^ per person; early-life source of drinking water (private water source vs public water source); and rural vs urban living at birth.

In MoBa, we also examined infant bed sharing (vs no bed sharing); similar data were unavailable in the ABIS study.

### Outcome

We identified IBD using linked data from the Swedish National Patient Register^10^ (ABIS study) and from the Norwegian Patient Registry^13^ (MoBa). These registers have a similar structure and contain nationwide diagnostic data on all inpatient and hospital-based outpatient care, including specialist care outside hospitals.^13,16^ Similar to previous studies,^17^ IBD was defined as having ≥ 2 recorded International Classification of Diseases–Tenth Revision (ICD-10) codes (Supplementary Table 3). This diagnostic algorithm for IBD has shown a positive predictive value of 93% for a clinical IBD diagnosis.^17,18^ We defined CD and UC using subtype-specific ICD-10 codes (Supplementary Table 3). Over the past 5 years of data capture, patients with a mix of both CD and UC diagnoses were classified as IBD–unclassified (IBD-U). Age at IBD diagnosis was defined as the age of the first recorded ICD-10 code, and data on IBD were recorded until December 31, 2020, in the ABIS study and December 31, 2021, in MoBa.

### Data on Covariates

Based on the literature,^5^ we preselected available adjustment variables that may affect the relationship between hygiene-related factors and IBD (illustrated in Supplementary Figure 2). Using questionnaire data at birth (ABIS study) and the 15th week of pregnancy (MoBa), we retrieved data on parental origin, education level, and maternal comorbidities (type 1 diabetes, autoimmune thyroid disease, and rheumatoid arthritis). In the ABIS study, parental origin was defined by the parent’s country of birth (Sweden or other country), and in MoBa, it was defined by the mother’s native language (ie, Norwegian or other languages). The Swedish Medical Birth Register^11^ and MBRN^14^ were used to collect data on maternal age at delivery. Information on the child’s sex, birth weight, gestational age, and delivery mode was obtained from the birth questionnaire (ABIS study) or the MBRN^14^ (MoBa). Data on parental IBD were collected from the birth questionnaire (ABIS study) and the Norwegian Patient Registry (MoBa).^13^ Questionnaire data on full breastfeeding were captured when the child was 12 (ABIS study) and 6 (MoBa) months of age. Finally, maternal smoking habits during pregnancy were retrieved from the at-birth questionnaire (ABIS study) and from questionnaires administered during pregnancy and by the child’s 6 months of age (MoBa).

### Statistical Analysis

We used the Cox proportional hazards model to estimate hazard ratios (HRs) and 95% confidence intervals (CIs) for IBD for each cohort separately. In a random-effects model^19^ we combined cohort-specific results into pooled HRs, our primary outcome measure. We used the Cochran Q test^20^ to examine between-group heterogeneity. The proportional hazards assumption was valid after graphically assessing the data, exploring interactions with time, and Schoenfeld residuals. Participants were followed from birth until IBD diagnosis or at the end of data capture (December 31, 2020 [ABIS study], and December 31, 2021 [MoBa]).

Analyses for the outcome of IBD included CD, UC, and IBD-U events. Subanalyses for the outcome of CD excluded participants with UC and IBD-U; subanalyses for the outcome of UC excluded participants with CD and IBD-U (due to limited cases, we chose a priori not to perform an IBD-U–specific analysis). All analyses were adjusted for the child’s sex, parental IBD, origin, education level, and maternal comorbidities. Secondary adjustment models also adjusted for delivery mode, birth weight, gestational age, full breastfeeding, maternal age at delivery, and smoking during pregnancy.

Statistical analyses were performed using R statistical software versions 4.1.3 and 4.2.2 (R Foundation for Statistical Computing) and SPSS version 28 (IBM). Cox proportional hazards analysis and checking assumptions were performed using the R packages survival and survminer. Pooled HRs and corresponding CIs were calculated using the DerSimonian and Laird random effects model^19^ with the R packages meta (v6.0-0) and metafor (v3.8-1). Complete-case analyses were performed, which ignored observations with incomplete covariate data. Because the associations evaluated in the current study are complementary, sharing a central biological hypothesis, we did not apply multiple testing corrections.^21^

Preplanned subanalyses considered the risk of childhood-onset IBD diagnosed before 18 years of age. To rule out the risk of reverse causation (ie, an IBD diagnosis affecting the exposure to hygiene-related factors), we repeated our main analyses after excluding 3 children with an IBD diagnosis <24 months of age. Any potential associations found were in post hoc analyses mutually adjusted for each other.

### Ethical Considerations

MoBa was approved by the Regional Committees for Medical and Health Research Ethics (#153328) and is regulated by the Norwegian Health and Registry Act. Approval was obtained for the ABIS study by the Research Ethics Committees of the Faculty of Health Sciences at Linköping University, Sweden (Ref. 1997/96287 and 2003/03-092) and the Medical Faculty of Lund University, Sweden (Dnr. 99227, Dnr. 99321).

## Results

We included data on 117 493 participants (ABIS study, n = 16 223; MoBa, n = 101 270) (Supplementary Figure 1). The mean age at the end of the follow-up was 22.3 years in the ABIS cohort and 16.4 years in the MoBa cohort (Table 1). In over 2 024 299 person-years (PYR) of follow-up, 451 participants developed IBD, corresponding to an incidence rate of 31 per 100 000 PYR in the ABIS cohort and 20 per 100 000 PYR in the MoBa cohort (Supplementary Table 4). Of the 451 participants diagnosed with IBD, 182 (40%) had CD and 152 (34%) had UC. Some 117 participants were defined as IBD-U based on a mix of subtype-specific diagnostic codes for IBD. Apart from a higher parental education level in the MoBa cohort, study characteristics did not differ between the two (Table 1). Background characteristics and IBD incidence rates were similar between participants in the study and those not included because of missing data, except for maternal smoking, which was somewhat more common in participants with missing data at 12 and 36 months of age (Supplementary Table 5).

**Table 1.** Descriptive characteristics of parents and children in the ABIS and MoBa cohorts

	ABIS		MoBa	
	All (n = 16 223)	IBD event (n = 113)	All (n = 101 270)	IBD event (n = 338)
Sex^a^				
Female	7821 (48.2)	52 (46.0)	49 400 (48.8)	146 (43.2)
Male	8402 (51.8)	61 (54.0)	51 870 (51.2)	192 (56.8)
Birth year^a^^,^^c^				
1997	1668 (10.3)	11 (9.7)	—	—
1998	8602 (53.0)	66 (58.4)	—	—
1999	5953 (36.7)	36 (31.9)	41 (0.0)	—
2000	—	—	1953 (1.9)	10 (3.0)
2001	—	—	3846 (3.8)	25 (7.4)
2002	—	—	8107 (8.0)	39 (11.5)
2003	—	—	11 910 (11.8)	52 (15.4)
2004	—	—	12 829 (12.7)	47 (13.9)
2005	—	—	14 907 (14.7)	49 (14.5)
2006	—	—	16 553 (16.3)	40 (11.8)
2007	—	—	15 269 (15.1)	34 (10.1)
2008	—	—	12 691 (12.5)	36 (10.7)
2009	—	—	3164 (3.1)	6 (1.8)
Age at the end of follow-up (years)^b^^,^^c^				
Mean (SD)	22.2 (1.0)	16.9 (3.7)	16.4 (2.2)	12.8 (3.8)
Median (IQR)	22.3 (21.8-22.8)	17.9 (15.0-19.6)	16.2 (14.7-18.1)	13.2 (10.7-15.5)
Age at IBD diagnosis, y^b^^,^^c^				
Mean (SD)	—	16.9 (3.7)	—	12.8 (3.8)
Median (IQR)	—	17.9 (15.0-19.6)	—	13.2 (10.7-15.5)
Range	—	2.6-22.7	—	3.1-21.9
Maternal origin^a^^,^^d^				
Swedish/ Norwegian	14 840 (91.5)	102 (90.3)	93 082 (91.9)	306 (90.5)
Other	1048 (6.5)	10 (8.8)	5658 (5.6)	17 (5.0)
Missing	335 (2.1)	1 (0.9)	2530 (2.6)	15 (4.4)
Paternal origin^a^				
Swedish	14 704 (90.6)	103 (91.2)	—	—
Other	1141 (7.0)	9 (8.0)	—	—
Missing	378 (2.3)	1 (0.9)	—	—
Maternal education level^a^^,^^e^				
0-11 y	5904 (36.4)	50 (44.2)	8029 (7.9)	37 (10.9)
12 y	4267 (26.3)	26 (23.0)	29 735 (29.4)	99 (29.3)
≥13 y	5669 (34.9)	36 (31.9)	62 997 (62.2)	199 (58.9)
* *Missing	383 (2.4)	1 (0.9)	509 (0.5)	3 (0.9)
Paternal education level^a^^,^^e^				
0-11 y	5373 (33.1)	35 (31.0)	10 462 (10.3)	44 (13.0)
12 y	6055 (37.3)	43 (38.1)	39 632 (39.1)	145 (42.9)
≥13 y	4177 (25.7)	33 (29.2)	47 789 (47.2)	140 (41.4)
Missing	618 (3.8)	2 (1.8)	3387 (3.3)	9 (2.7)
Parental IBD^a^^,^^c^^,^^f^				
Yes	195 (1.2)	5 (4.4)	2325 (2.3)	9 (2.7)
Missing	0	0	0	0
Maternal comorbidities^a^^,^^g^				
Yes	568 (3.5)	4 (3.5)	4072 (4.0)	13 (3.8)
Missing	0	0	0	0
Maternal smoking^a^				
Yes	1760 (10.8)	13 (11.6)	9597 (9.5)	47 (13.9)
Missing	380 (2.3)	1 (0.9)	1587 (1.6)	7 (2.1)
Maternal age at delivery, y^a^^,^^h^				
<25	2551 (15.7)	15 (13.3)	11 071 (10.9)	34 (10.1)
25-34	11 440 (70.5)	83 (73.5)	72 431 (71.5)	142 (71.6)
35-44	1972 (12.1)	14 (12.4)	17 714 (17.5)	63 (18.4)
Missing	260 (1.6)	1 (0.9)	54 (0.1)	0
Delivery mode^a^^,^^b^				
Vaginal	13 040 (80.4)	91 (80.5)	86 028 (85.0)	275 (81.4)
Caesarean	1866 (11.5)	15 (13.3)	15 242 (15.0)	63 (18.6)
Missing	1317 (8.1)	7 (6.2)	0 (0.0)	0 (0.0)
Birth weight, g^a^^,^^b^^,^^i^				
Mean (SD)	3575 (552)	3551 (558)	3567 (589)	3648 (598)
Median (IQR)	3580 (3240-3930)	3600 (3305-3850)	3600 (3250-3940)	3670 (3250-4065)
Range	575-6850	1050-5000	410-6300	1195-5390
Missing	193 (1.2)	2 (1.8)	63 (0.1)	1 (0.3)
Gestational age^a^^,^^j^				
Mean (SD)	39.7 (1.8)	39.6 (1.7)	39.4 (1.9)	39.5 (1.8)
Median (IQR)	40.0 (39.0-41.0)	40.0 (39.0-41.0)	40.0 (39.0-41.0)	40.0 (39.0-41.0)
Range	25.0-43.0	32.0-42.0	23.0-45.0	28.0-43.0
* *Missing	333 (2.1)	2 (1.8)	459 (0.4)	1 (0.3)
Full breastfeeding^a^				
<4 mo	2941 (18.1)	25 (22.1)	36 017 (35.6)	115 (43.0)
4-6 mo	3629 (22.4)	24 (21.2)	39 369 (38.9)	134 (39.6)
≥6 mo	1587 (9.8)	15 (13.3)	12 043 (11.9)	49 (14.6)
* *Missing	8066 (49.7)	49 (43.4)	13 841 (13.7)	40 (11.8)
Crohn’s disease^b^^,^^c^	40 (0.5)	40 (35.4)	142 (0.1)	142 (42.0)
Ulcerative colitis^b^^,^^c^	57 (0.5)	57 (50.4)	95 (0.1)	95 (28.1)

Values are n (%), unless otherwise indicated.

Abbreviations: ABIS, All Babies in Southeast Sweden; IBD, inflammatory bowel disease; IQR, interquartile range; MoBa, Norwegian Mother, Father and Child Cohort Study.

^a^Data from questionnaires.

^b^Data from the Swedish National Patient Register (ABIS).

^c^Data from the Medical Birth Registry of Norway/the Norwegian Patient Registry (MoBa).

^d^Mother tongue (MoBa)/mother’s country of birth (ABIS).

^e^Education at time of birth.

^f^≥1 parent with IBD.

^g^Type 1 diabetes (insulin-treated diabetes before or during pregnancy [MoBa] or type 1 diabetes/insulin-treated diabetes [ABIS]), autoimmune thyroid disease, and/or rheumatoid arthritis.

^h^<15 years was defined as missing in ABIS (not applicable in MoBa) and >44 years were changed to missing in both cohorts.

^i^<270 or >6999 g were changed to missing.

^j^<22 or >45 weeks were changed to missing.

### Early-Life Hygiene-Related Exposures and Later IBD

**Figure 1.**
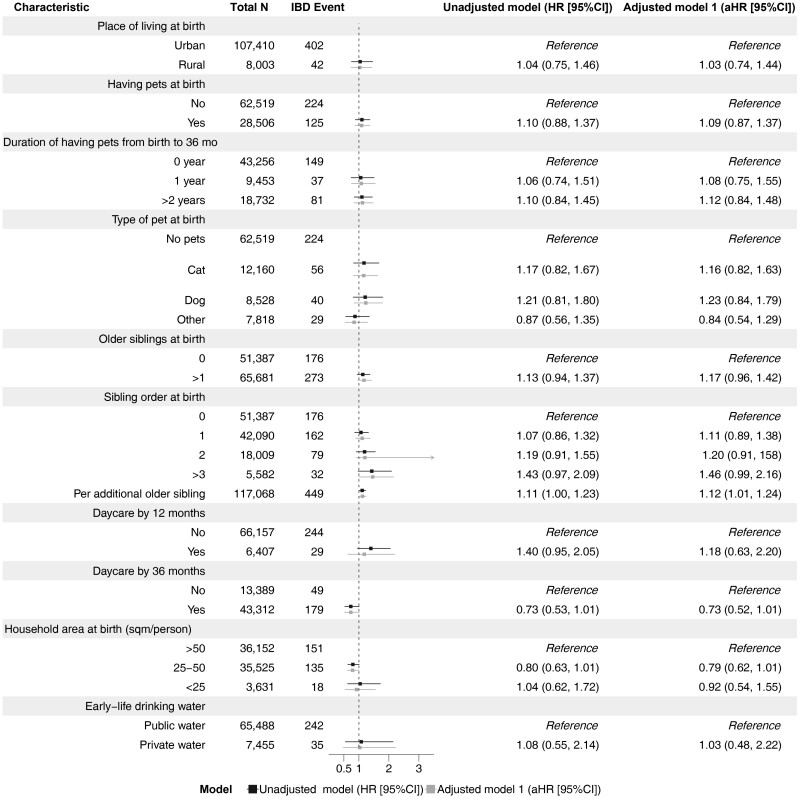
Pooled estimates of hygiene-related exposures of early life and later risk of inflammatory bowel disease (IBD) in the All Babies in Southeast Sweden and the Norwegian Mother, Father and Child Cohort Study cohorts. Adjusted model 1 (primary model): sex, parental IBD, parental origin, parental education level, and maternal comorbidities (type 1 diabetes, autoimmune thyroid disease, and/or rheumatoid arthritis). CI, confidence interval; HR, hazard ratio.

**Figure 2.**
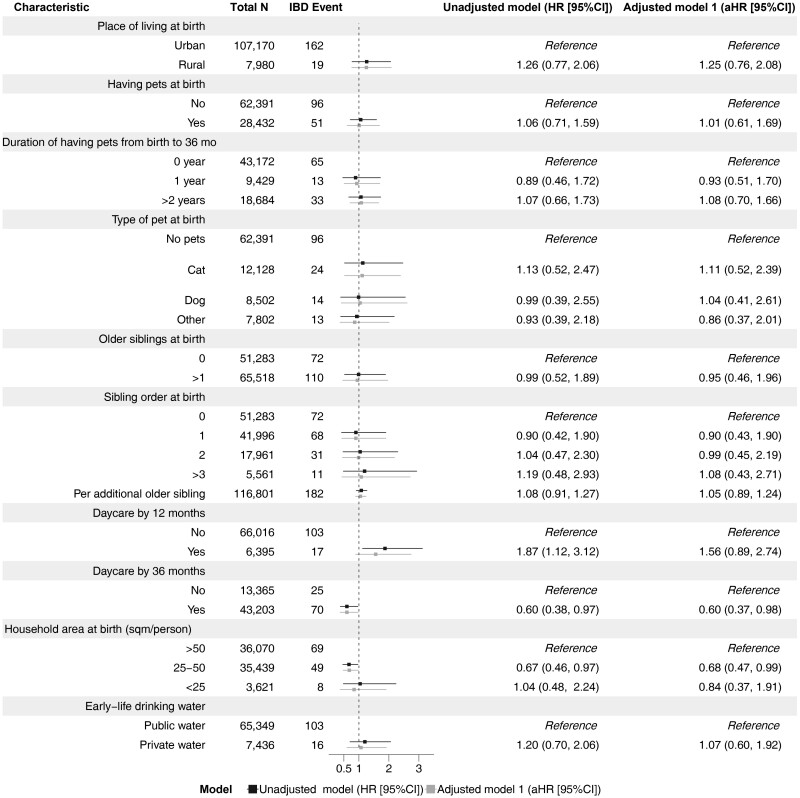
Pooled estimates of hygiene-related exposures of early life and later risk of Crohn’s disease in the All Babies in Southeast Sweden and the Norwegian Mother, Father and Child Cohort Study cohorts. Adjusted model 1 (primary model): sex, parental inflammatory bowel disease (IBD), parental origin, parental education level, and maternal comorbidities (type 1 diabetes, autoimmune thyroid disease, and/or rheumatoid arthritis). CI, confidence interval; HR, hazard ratio.

**Figure 3.**
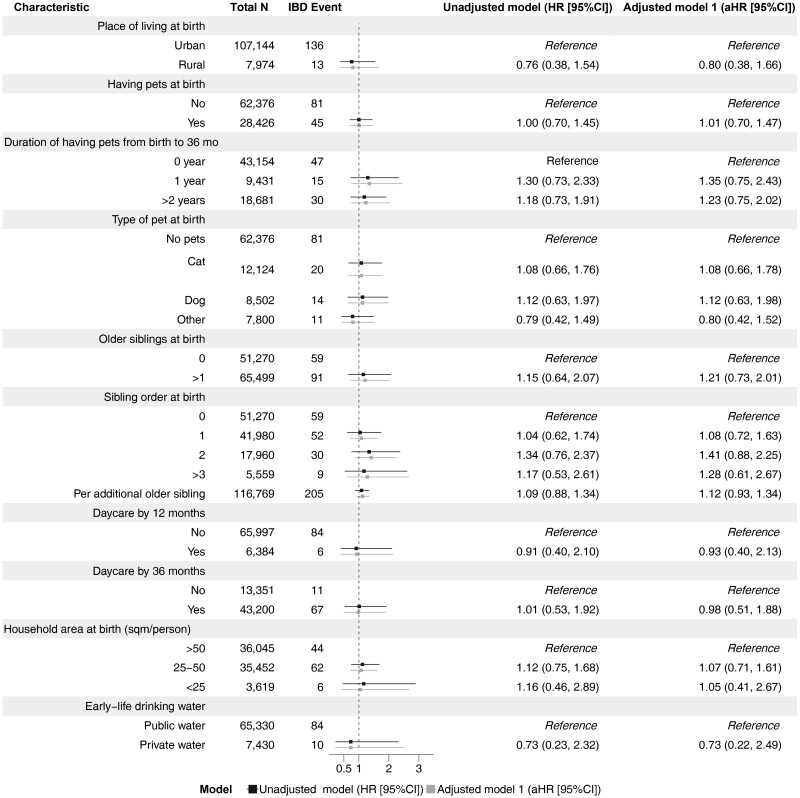
Pooled estimates of hygiene-related exposures of early life and later risk of ulcerative colitis in the All Babies in Southeast Sweden and the Norwegian Mother, Father and Child Cohort Study cohorts. Adjusted model 1 (primary model): sex, parental inflammatory bowel disease (IBD), parental origin, parental education level, and maternal comorbidities (type 1 diabetes, autoimmune thyroid disease, and/or rheumatoid arthritis). CI, confidence interval; HR, hazard ratio.

Children attending daycare by age 36 months (n = 43 312 of 56 701) vs not attending were associated with a reduced IBD risk, particularly CD (IBD: pooled adjusted HR [aHR], 0.73; 95% CI, 0.52-1.01; CD: pooled aHR, 0.60; 95% CI, 0.37-0.98) (Figures 1 and 2). These associations were essentially unchanged when adjusting for perinatal factors (delivery mode, birth weight, gestational age, maternal smoking, maternal age, and breastfeeding) (Supplementary Table 6). However, daycare attendance by 12 months vs no daycare attendance was not linked to later IBD (Figure 1). Children having 1 or more siblings (n = 65 681 of 117 493) were at increased risk of IBD (pooled aHR, 1.17; 95% CI, 0.96-1.42; per older sibling, pooled aHR, 1.12; 95% CI, 1.01-1.24) (Figure 1). Results of subtype-specific analyses are presented in Figure 2 (CD) and Figure 3 (UC).

Approximately 1 in 3 participants reported having a pet at birth (n = 28 506 of 91 452; Figure 1); Having any pet vs not having a pet was not associated with later IBD (pooled aHR, 1.09; 95% CI, 0.87-1.37), nor was there an association between the type of pet and IBD (Figure 1). Estimates for having any pet were unchanged in analyses adjusting for perinatal characteristics (Supplementary Table 6). Pet exposure in early life was also not linked to the risk of UC or CD (Figures 2 and 3). At birth, household crowding was reported for 4.6% (n = 3631 of 78 987) of the participants (Figure 1). Compared with >50 m^2^ per person, household crowding was not linked to later IBD (pooled aHR, 0.92; 95% CI, 0.54-1.55). In addition, pooled aHRs for IBD according to early-life drinking water approximated 1 (Figures 1-3).

Of 117 493 participants, 8003 (6.8%) had a rural residence early in life. Rural vs urban living in early life was not associated with later IBD (pooled aHR, 1.03; 95% CI, 0.74-1.44) (Figure 1), CD (pooled aHR, 1.25; 95% CI, 0.76-2.08) (Figure 2), and UC (pooled aHR, 0.80; 95% CI, 0.38-1.66) (Figure 3). Our cohort-specific analyses revealed no association between early-life rural living and IBD risk (ABIS study: aHR, 0.87; 95% CI, 0.54-1.40; MoBa: aHR, 1.22; 95% CI, 0.76-1.97) (Supplementary Table 7). Infant bed sharing was reported in 20% of MoBa participants (n = 16 719 of 75 229); the aHR was significant for IBD (aHR, 1.35; 95% CI, 1.01-1.79) and UC (aHR, 1.67; 95% CI, 1.01-2.78), but not for CD (aHR, 1.21; 95% CI = 0.78-1.88), and did not change after adjusting for perinatal factors (Supplementary Tables 7-9). Data on bed sharing were not available in the ABIS cohort.

### Preplanned Subanalyses

Of 451 participants with IBD, 381 (84.5%) were diagnosed with the disease by 18 years of age. Restricting follow-up to <18 years of age, associations between hygiene-related exposures and childhood-onset IBD, CD, and UC were similar compared with our main analyses (Supplementary Table 10). To rule out the risk of reverse causation, we reran our main analysis after excluding 3 children diagnosed with IBD <24 months of age. This analysis yielded unchanged estimates compared with our main analysis (data not shown). Mutual adjustment across exposures, eg, adding siblings as a covariate in analyses of daycare, revealed essentially unchanged risk estimates (data not shown).

## Discussion

Using birth cohort data from 2 Scandinavian high-income countries, participants with older siblings were associated with a modestly increased risk of later IBD. In contrast, children who start daycare before they turn 36 months of age have a reduced risk of later IBD compared with children without daycare attendance. HRs for most other early-life hygiene-related exposures, including rural vs urban living and having pets, approximated 1. The generalizability of our results to low- and middle-income countries need further study.

The hygiene hypothesis suggests that a lack of microbial antigen exposure associated with improved hygiene may increase the risk of immune-mediated diseases, including IBD.^22^ Animal studies have linked improved hygiene to decreased microbiota diversity and promotion of a proinflammatory T helper type 2–mediated immune response.^23^ Moreover, hygiene exposures during early childhood, a period critical for the developing intestinal immune system, microbiome, and mucosal-bacterial interactions, may play a vital role in IBD development.

In accordance with the hygiene hypothesis, daycare attendance by 36 months of age seemed to be associated with a reduced risk of IBD (pooled aHR, 0.73; 95% CI, 0.52-1.01), particularly CD (pooled aHR, 0.60; 95% CI, 0.37-0.98). This subtype-specific finding is supported by previous work suggestion that dysbiosis may be greater in CD compared with UC.^24^ However, due to the wide 95% CIs and the likelihood for a patient to change IBD subtype diagnosis during follow-up,^25^ the difference should be cautiously interpreted. Daycare attendance has been linked to a reduced risk of allergic disease^26^ but has been examined little in the context of IBD. The only previous study in this field found no overall association between daycare attendance and IBD, except for an increased risk of later CD in children with daycare attendance <6 months of age.^27^ While we did not find an association between daycare attendance by 12 months of age and later IBD (pooled aHR, 1.18; 95% CI, 0.63-2.20), early daycare attendance was relatively uncommon in our cohort (8.7% [n = 6407 of 73 627]) and prevented us from ruling out effect sizes of moderate magnitude.

In contrast to daycare supporting the hygiene hypothesis, we found a lower sibling order to be modestly associated with increased risk for IBD, in which every additional sibling increased the risk. In contrast to other countries,^28^ Swedish and Norwegian households with children, particularly older children, have a higher income compared with households without children.^29,30^ This indicates that larger families living in Sweden and Norway have higher household income and socioeconomic status and therefore increased sanitary living, supporting the hygiene hypothesis specifically to Scandinavia. In previous studies, a higher number of siblings have been associated with a reduced risk of IBD, most notably for CD.^31,32^ A recent systematic review and meta-analysis reported that 3 of 12 studies found an association between household size or birth order and later IBD risk.^5^ However, these 3 studies were all case-control studies and thus liable to recall bias.

Rural living in childhood, which has been shown to influence the gut microbiome, has been suggested to have a protective role in IBD development compared with urban residency.^33^ One population-based cohort study in Canada found a negative association between rurality at 1 to 5 years of age and later IBD.^7^ However, similar to a recent register-based Swedish study,^34^ we found no association between rural vs urban living in early life and later IBD. Nevertheless, considering the relatively low participation rate of 41% in the MoBa cohort, we cannot rule out that rural living may be underrepresented in our study. Also, rurality may have different meanings across different cultural settings. For example, the effect of rural living on the risk of CD differs between adults living in Israel and China, along with country-specific dietary signals and microbial compositions.^35^

Infant bed sharing has been linked to a reduced risk of IBD,^31^ particularly CD.^36^ In the MoBa cohort, we found that bed sharing was associated with an increased risk of IBD. However, these results should be interpreted cautiously given that bed sharing was only possible to analyze in the MoBa cohort and was relatively uncommon (only reported for 20%) with a lower-bound 95% CI just above 1 (1.01-1.79). While primarily retrospective data have associated household crowding,^27^ private drinking water,^37^ and having pets^36^ with a reduced risk of IBD, in this prospective study, as in most other works,^5^ these exposures were not significantly associated with later IBD.

Evidence of the association between hygiene-related exposures and IBD is inconsistent.^5^ This inconsistency may partly be affected by the heterogeneity of study populations, comparison groups, and exposure definitions. Because most studies have been retrospective case-control studies, results may also have been influenced by the risk of recall bias, selection bias, and even reverse causation. While the inconsistency in the direction and magnitude of associations on IBD risk may argue against a causal explanation for the findings, these equivocal relationships may also reflect different proxies that warrant further investigation.

### Strengths and Limitations

This study is one of the first prospective studies to investigate the association between early-life hygiene-related exposures and later IBD. The 2 Scandinavian birth cohorts have close similarities in their structure and method of data collection, which enabled us to test the consistency of results across cohorts. Data from 2 studies are important because, from a societal and ethical perspective, only consistent findings from prospective cohorts would motivate the initiation of long-term and costly preventive intervention studies against IBD. Including data on >117 000 children, followed from birth throughout childhood, allowed us to perform important subanalyses, such as restriction to childhood-onset IBD. Compared with studies based on tertiary care centers,^27,32^ our population-based design reduces the risk of selection bias. Linkage to nationwide patient registers^10,13^ reduced the impact of the loss to follow-up. Our analyses of children with vs without follow-up for up to 36 months of age argued against selective dropout. Finally, combined data from comprehensive questionnaires and national registries enabled us to adjust for several potential confounders, including breastfeeding duration and parental smoking habits.

Compared with retrospective studies, our prospectively collected data may reduce the risk of recall bias. However, because most data on hygiene conditions are not recorded in any patient registers and this study relies on self-reported data, we cannot exclude the possibility that erroneously recall or social desirability bias may have affected our results. The participation rate of 41% in MoBa (79% in the ABIS study) may raise questions about the representativeness of the cohort and may have introduced bias. The lower participation rate in the MoBa cohort is in line with other nationwide cohorts^38^ but may raise questions about the representativeness of the cohort. Compared with all Norwegian mothers, MoBa participants were older, were less likely to smoke during pregnancy,^39^ and had higher education.^40^ Accordingly, our results may be less generalizable to underrepresented populations, such as families with low socioeconomic status or immigrants. Also, we cannot rule out that the predominance of including higher-educated mothers, may have introduced bias by self-selection.

As in any observation study, we cannot rule out the possibility that residual confounding may have influenced the results, and one should interpret the presented findings cautiously (eg, we were unable to account for antibiotic use and childhood infections and could therefore not rule out that any association found may have been mediated by such exposures). We only included data on clinical IBD diagnoses, rather than on preclinical disease manifestations that, although very rare in early life, might have influenced bed-sharing habits and the timing of daycare attendance. We also lacked genetic data, which prevented us from studying whether the effect of hygiene-related exposures may vary across genetic risk groups of IBD. In addition, we could not investigate the influence of early-life hygiene-related exposures on the developing gut microbiome.^6^ We suspect that alterations (dysbiosis) may play a key role in IBD development.^41^ Missing data, particularly for exposures captured at 3 years, may be related to internal attrition and introduce type II error. However, as presented in Supplementary Table 5, missingness seemed to not be related to the risk of IBD.

Finally, because our findings originated from 2 high-income countries with high incidences of IBD, we may not be able to generalize to developing countries with different living conditions. For example, our definition of household crowding and daycare may not be applicable to other populations outside Western countries. The cutoff of 25 m^2^ per person was based on Norwegian standards^15^ and may not be classified as high household crowding in other countries, such as India, a country with increasing incidence of IBD and a socioeconomic setting highly distinct from Scandinavian countries.^42^ Because a majority of our study population had household size >50 sqm/person, and high parental education level, our findings should be cautiously interpreted to countries with greater variation of socioeconomic status.^43,44^ Hence, it is possible that our findings may foremost pertain to countries of similar socioeconomic status.

Using prospectively collected birth cohort data from 2 high-income Scandinavian countries, we found some, but not all, hygiene-related exposures in early life to be associated with IBD risk. The found inconsistency in the direction and magnitude of associations on IBD risk warrants further research. Finally, the generalizability of our findings to low- or middle-income countries is unknown.

## Supplementary data

Supplementary data is available at *Inflammatory Bowel Diseases* online.

izad257_suppl_Supplementary_Material

## Data Availability

The data collected for this article will be shared on reasonable request to the study’s principal investigator K.M.
