# Supplementary material for: Early-Life Hygiene-Related Factors and Risk of Inflammatory Bowel Disease: A Scandinavian Birth Cohort Study
Source: Inflamm Bowel Dis. 2023 Oct 31;30(10):1820–30. doi: 10.1093/ibd/izad257 (PMC11447116; doi:10.1093/ibd/izad257)
Supplement: izad257_suppl_Supplementary_Material [file izad257_suppl_supplementary_material.docx]

**Supplemental Data**

*Supplement to: Guo A et al. Early-life Hygiene-related Factors and Risk of Inflammatory Bowel Disease: A Scandinavian Birth Cohort Study*

Table of Content

[Tables 2](#_Toc142574932)

[**Supplementary Table 1**. Summary table of studies on early-life hygiene-related exposures and risk of Crohn’s disease and ulcerative colitis 3](#_Toc142574933)

[**Description of hygiene-related exposures** 5](#_Toc142574934)

[**Supplementary Table 2**. Harmonization of hygiene-related exposure variables in the ABIS and MoBa cohorts 7](#_Toc142574935)

[**Supplementary Table 3.** Definition of inflammatory bowel disease^a^ 11](#_Toc142574936)

[**Supplementary Table 4.** Prevalence and incidence with 95% CI for inflammatory bowel disease, Crohn’s disease, and ulcerative colitis in the ABIS and MoBa cohorts 12](#_Toc142574937)

[**Supplementary Table 5.** Characteristics of included and excluded participants in the ABIS and MoBa cohorts 13](#_Toc142574938)

[**Supplementary Table 6.** Pooled hazard ratios for the risk of inflammatory bowel disease, Crohn’s disease and ulcerative colitis adjusted for perinatal characteristics^a^ 14](#_Toc142574939)

[**Supplementary Table 7.** Hygiene-related exposures of early-life and later risk of inflammatory bowel disease in the ABIS and MoBa cohorts 15](#_Toc142574940)

[**Supplementary Table 8.** Hygiene-related exposures of early-life and later risk of Crohn’s disease in the ABIS and MoBa cohorts 19](#_Toc142574941)

[**Supplementary Table 9.** Hygiene-related exposures of early-life and later risk of ulcerative colitis in the ABIS and MoBa cohorts 23](#_Toc142574942)

[**Supplementary Table 10.** Pooled hazard ratios of inflammatory bowel disease by age <18 years 27](#_Toc142574943)

[**Supplementary Table 11.** STROBE Checklist for cohort studies 28](#_Toc142574944)

[Figures 31](#_Toc142574945)

[**Supplementary Figure 1** 31](#_Toc142574946)

[**Supplementary Figure 2** 32](#_Toc142574947)

[References 33](#_Toc142574948)

# **Tables**

**Literature review**

On November 30, 2022 we searched PubMed (https://pubmed.ncbi.nlm.nih.gov) for publications examining hygiene-related exposures of early life as risk factors for inflammatory bowel disease (IBD). The search string presented below is similar to the one used in a recent systematic review on environmental risk factors for IBD.^1^ Inclusion criteria were full-text English articles published since the year 2000 reporting original data on children. Finally, the reference lists of relevant studies were screened by publication titles for additional relevant articles. The literature search was conducted by AG with the support of a professional librarian. Eligibility screening and data extraction were performed by AG. Total 184 unique articles were identified and first screened by titles and abstracts, and second by full-text review. Eventually, six studies were deemed relevant from which data were extracted (Supplementary Table 1).

(("paediatrics"(All Fields) OR "pediatrics"(MeSH Terms) OR "pediatrics"(All Fields) OR "paediatric"(All Fields) OR "pediatric"(All Fields)) AND ("inflammatory bowel diseases"(MeSH Terms) OR ("inflammatory"(All Fields) AND "bowel"(All Fields) AND "diseases"(All Fields)) OR "inflammatory bowel diseases"(All Fields) OR ("inflammatory"(All Fields) AND "bowel"(All Fields) AND "disease"(All Fields)) OR "inflammatory bowel disease"(All Fields)) AND ((("animals, domestic"(MeSH Terms) OR ("animals"(All Fields) AND "domestic"(All Fields)) OR "domestic animals"(All Fields) OR "domestic"(All Fields) OR "domestically"(All Fields) OR "domesticate"(All Fields) OR "domesticated"(All Fields) OR "domesticates"(All Fields) OR "domesticating"(All Fields) OR "domestication"(MeSH Terms) OR "domestication"(All Fields) OR "domestications"(All Fields) OR "domestics"(All Fields)) AND "animal*"(All Fields)) OR (("farms"(MeSH Terms) OR "farms"(All Fields) OR "farm"(All Fields)) AND "animal*"(All Fields)) OR ("animals, domestic"(MeSH Terms) OR ("animals"(All Fields) AND "domestic"(All Fields)) OR "domestic animals"(All Fields) OR "domestic"(All Fields) OR "domestically"(All Fields) OR "domesticate"(All Fields) OR "domesticated"(All Fields) OR "domesticates"(All Fields) OR "domesticating"(All Fields) OR "domestication"(MeSH Terms) OR "domestication"(All Fields) OR "domestications"(All Fields) OR "domestics"(All Fields)) OR ("farms"(MeSH Terms) OR "farms"(All Fields) OR "farm"(All Fields)) OR "pet"(All Fields) OR ("pets"(MeSH Terms) OR "pets"(All Fields)) OR ("dogs"(MeSH Terms) OR "dogs"(All Fields) OR "dog"(All Fields)) OR "cat"(All Fields) OR (((("urban"(All Fields) OR "urbanicity"(All Fields) OR "urbanism"(All Fields) OR "urbanity"(All Fields) OR "urbanization"(MeSH Terms) OR "urbanization"(All Fields) OR "urbanizations"(All Fields) OR "urbanize"(All Fields) OR "urbanized"(All Fields) OR "urbanizes"(All Fields) OR "urbanizing"(All Fields) OR "urbans"(All Fields)) AND ("hygiene"(MeSH Terms) OR "hygiene"(All Fields) OR "hygienic"(All Fields) OR "hygienical"(All Fields) OR "hygienically"(All Fields) OR "hygienics"(All Fields) OR "hygienization"(All Fields))) OR (("rural"(All Fields) OR "ruralities"(All Fields) OR "rurality"(All Fields) OR "rurally"(All Fields) OR "ruralness"(All Fields) OR "rurals"(All Fields)) AND ("hygiene"(MeSH Terms) OR "hygiene"(All Fields) OR "hygienic"(All Fields) OR "hygienical"(All Fields) OR "hygienically"(All Fields) OR "hygienics"(All Fields) OR "hygienization"(All Fields))) OR ("rural population"(MeSH Terms) OR ("rural"(All Fields) AND "population"(All Fields)) OR "rural population"(All Fields)) OR ("urban population"(MeSH Terms) OR ("urban"(All Fields) AND "population"(All Fields)) OR "urban population"(All Fields)) OR ("urban"(All Fields) OR "urbanicity"(All Fields) OR "urbanism"(All Fields) OR "urbanity"(All Fields) OR "urbanization"(MeSH Terms) OR "urbanization"(All Fields) OR "urbanizations"(All Fields) OR "urbanize"(All Fields) OR "urbanized"(All Fields) OR "urbanizes"(All Fields) OR "urbanizing"(All Fields) OR "urbans"(All Fields))) AND ("rural"(All Fields) OR "ruralities"(All Fields) OR "rurality"(All Fields) OR "rurally"(All Fields) OR "ruralness"(All Fields) OR "rurals"(All Fields))) OR ("birth order"(MeSH Terms) OR ("birth"(All Fields) AND "order"(All Fields)) OR "birth order"(All Fields) OR "sibling*"(All Fields) OR "siblingship*"(All Fields)) OR ("child day care centers"(MeSH Terms) OR ("child"(All Fields) AND "day"(All Fields) AND "care"(All Fields) AND "centers"(All Fields)) OR "child day care centers"(All Fields) OR "daycare"(All Fields) OR "daycares"(All Fields) OR "childminder*"(All Fields) OR ("nurseries, infant"(MeSH Terms) OR ("nurseries"(All Fields) AND "infant"(All Fields)) OR "infant nurseries"(All Fields) OR "nurseries"(All Fields) OR "nursery"(All Fields))) OR ("water"(MeSH Terms) OR "water"(All Fields) OR "drinking water"(MeSH Terms) OR ("drinking"(All Fields) AND "water"(All Fields)) OR "drinking water"(All Fields) OR "watering"(All Fields) OR "water s"(All Fields) OR "watered"(All Fields) OR "waterer"(All Fields) OR "waterers"(All Fields) OR "waterings"(All Fields) OR "waters"(All Fields)) OR ("bedshare"(All Fields) OR "bedshared"(All Fields) OR "bedsharing"(All Fields) OR (("complications"(MeSH Subheading) OR "complications"(All Fields) OR "co"(All Fields)) AND "sleep*"(All Fields)) OR (("beds"(MeSH Terms) OR "beds"(All Fields) OR "bed"(All Fields)) AND ("share"(All Fields) OR "shared"(All Fields) OR "shares"(All Fields) OR "sharing"(All Fields) OR "sharings"(All Fields)))) OR ("crowd*"(All Fields) OR ("population density"(MeSH Terms) OR ("population"(All Fields) AND "density"(All Fields)) OR "population density"(All Fields)) OR (("populate"(All Fields) OR "populated"(All Fields) OR "populates"(All Fields) OR "populating"(All Fields) OR "population"(MeSH Terms) OR "population"(All Fields) OR "population groups"(MeSH Terms) OR ("population"(All Fields) AND "groups"(All Fields)) OR "population groups"(All Fields) OR "populations"(All Fields) OR "population s"(All Fields) OR "populational"(All Fields) OR "populous"(All Fields)) AND ("abundance"(All Fields) OR "abundances"(All Fields) OR "abundancies"(All Fields) OR "abundancy"(All Fields) OR "abundant"(All Fields))) OR "populationsize"(All Fields) OR ("family characteristics"(MeSH Terms) OR ("family"(All Fields) AND "characteristics"(All Fields)) OR "family characteristics"(All Fields) OR ("family"(All Fields) AND "size"(All Fields)) OR "family size"(All Fields)) OR (("family characteristics"(MeSH Terms) OR ("family"(All Fields) AND "characteristics"(All Fields)) OR "family characteristics"(All Fields) OR "household"(All Fields) OR "households"(All Fields) OR "household s"(All Fields) OR "householder"(All Fields) OR "householder s"(All Fields) OR "householders"(All Fields)) AND "crowd*"(All Fields))))) AND ((english(Filter)) AND (infant(Filter) OR newborn(Filter) OR allinfant(Filter) OR preschoolchild(Filter) OR child(Filter)) AND (2000:2022(pdat)))

## **Supplementary Table 1**. Summary table of studies on early-life hygiene-related exposures and risk of Crohn’s disease and ulcerative colitis

| **Author, year (country)** | **Study design** | **IBD event**  **(age)** | **Hygiene exposure (definition)** | **Crohn’s disease**  **Risk estimate (95% CI)** | | **Ulcerative colitis**  **Risk estimate (95% CI)** | |
| --- | --- | --- | --- | --- | --- | --- | --- |
|  |  |  |  | Crude model | Adjusted model | Crude model | Adjusted model |
| Amre, 2006 (Canada)^2 a^ | Case-control study | 194  (0-20 years) | Daycare attendance  (never (ref.) vs. ever) | OR 1.0 (0.7-1.5) |  |  |  |
|  |  |  | Daycare attendance (age-periods when attended)^b^  Between birth and 6 months |  | aOR 4.5 (1.4-13.7) |  |  |
|  |  |  | Between 7 and 12 months Between 12 and 36 months  Age 48 months and above |  | aOR 1.5 (0.8-2.9) aOR 1.1 (0.6-1.8) aOR 0.9 (0.5-1-6) |  |  |
|  |  |  | Bed-sharing  (never/sometimes (ref.) vs. always) | OR 1.0 (0.4-2.4) |  |  |  |
|  |  |  | Pets  (never/sometimes (ref.) vs. always) | OR 2.1 (1.3-3.3) | aOR 2.0 (0.90-4.50) |  |  |
|  |  |  | Household crowding (high crowding (ref.) vs. lesser crowding) |  | aOR 0.33 (0.13-0.84) |  |  |
| Baron, 2005 (France)^3 a^ | Case-control study | 282  (0-17 years) | Bedroom sharing  (not sharing bedroom (ref.) vs. sharing bed room) | OR 1.6 (1.0-2.4) | aOR 3.4 (1.5-7.9) | OR 3.4 (1.5-7.9) | aOR 7.1 (1.9-27.4) |
|  |  |  | Water  (not drinking tap water (ref.) drinking tap water) | OR 0.5 (0.3-0.8) | aOR 0.6 (0.3-1.0) |  |  |
| Jakobsen, 2013 (Denmark)^4^ | Case-control study | 118  (0-15 years) | Bedroom sharing (no (ref.) vs. yes) | OR 1.3 (0.6–2.6) | aOR 3.6 (1.3–9.4) | Not significant | Not significant |
| Lee, 2022 (Malaysia)^5^ ^a^ | Case-control study | 70  (0-18 years) | Attending the same nursery at the same time with siblings (no (ref.) vs. yes) | OR 1.14 (0.48-2.72) |  | OR 2.68 (1.06-6.82) | aOR 1.80 (0.44-7.32) |
|  |  |  | Any sibling  (no (ref.) vs. yes) | OR 0.90 (0.31-2.66) |  | OR 2.01 (0.52-7.77) |  |
|  |  |  | Number of siblings (median) | OR 0.93 (0.70-1.24) |  | OR 1.18 (0.85-1.63) |  |
|  |  |  | Bottled water for drinking and cooking  (no (ref.) vs. yes) | OR 0.32 (0.14-0.75) | aOR 0.68 (0.23-2.02) | OR 0.22 (0.07-0.71) | aOR 0.17 (0.04-0.78) |
|  |  |  | Well water for drinking and cooking  (no (ref.) vs. yes) | OR 1.00 (0.00-0.00) |  | OR 1.00 (0.00-0.00) |  |
|  |  |  | Aquarium fishes/turtles  (no (ref.) vs. yes) | OR 2.66 (0.76-9.37) |  | OR 2.72 (0.76-9.73) |  |
|  |  |  | Dog  (no (ref.) vs. yes) | OR 2.05 (0.75-5.57) |  | OR 0.87 (0.25-3.09) |  |
|  |  |  | Cat  (no (ref.) vs. yes) | OR 0.91 (0.32-2.61) |  | OR 0.34 (0.09-1.27) |  |
|  |  |  | Bird  (no (ref.) vs. yes) | OR 3.17 (0.51-19.85) |  | OR 1.00 (0.09-1.46) |  |
|  |  |  | Sharing bedroom with family member  (no (ref.) vs. yes) | OR 1.27 (0.23-6.86) |  | OR 0.64 (0.14-3.07) |  |
| Radon, 2007 (Germany)^6^ | Case-control study | 747  (6-18 years) | Urban living  (rural living (ref.) vs. urban living) | OR 1.5 (1.1–2.0)^c^ | aOR: 1.5 (1.1–2.0) | OR 1.6 (1.2–2.2)^c^ | aOR 1.5 (1.1–2.1) |
|  |  |  | Regular contact with farm animals >1 week during first year of life  (no (ref.) vs. yes) | OR 0.5 (0.3–0.8)^c^ | aOR 0.5 (0.3–0.9) | OR 0.3 (0.2–0.6)^c^ | aOR 0.4 (0.2–0.8) |
|  |  |  | Regular contact with pets >1 week during first year of life  (no (ref.) vs. yes) | OR 0.8 (0.6-1.1)^c^ | aOR 1.0 (0.8-1.3) | OR 0.7 (0.5-0.9)^c^ | aOR 0.9 (0.6-1.2) |
|  |  |  | Having older sibling  (0 (ref.) vs. >2) | OR 0.5 (0.4-0.8)^c^ | aOR 0.6 (0.4-0.8) | OR 0.8 (0.6-1.2)^c^ | aOR 0.9 (0.6-1.2) |
| Strisciuglio, 2017 (Italy)^7 a^ | Case-control study | 467  (0-18 years) | Bed-sharing (sharing bed or bedroom with other family member)  (no (ref.) vs. yes) | OR 0.3 (0.2-0.6) | aOR: 0.2 (0.1-0.6) |  |  |
|  |  |  | Number siblings  (>2 (ref.) vs. <2) | OR 2.1 (1.3-3.6) | aOR 2.8 (1.5-5.2) | OR 2.0 (1.3-3.1) | aOR 2.0 (1.1-3.6) |
|  |  |  | Pets  (no (ref.) vs. yes) | OR 0.4 (0.2-0.7) | aOR 0.3 (0.1-0.7) | OR 0.5 (0.3-0.9) | aOR 0.4 (0.2-0.8) |

^a^Estimates not reported in the original paper. ^b^Reference category not reported. ^c^Adjusted for age and sex. CD, Crohn’s disease; CI, confidence interval; OR, odds ratio; UC, ulcerative colitis.

## **Description of hygiene-related exposures**

The text below describes the definitions of hygiene-related exposures with details provided in Supplementary Table 2.

*Pets*

Similar to other studies,^2, 7^ we defined pet exposure as having pets at home (any pet vs. no pet), i.e., we did not consider pet exposure outside the home. Data were collected from self-administered questionnaires at birth (ABIS) and when the child was 6 months of age (MoBa). The type of pet was categorized as dog, cat, and other animal (also including multiple pets). We also analyzed the duration of pet exposure by 36 months of age based on the number of questionnaires administered in each cohort until that age: 0 years (reporting no pets), 1 year (reporting having pets in one questionnaire), ≥2 years (reporting having pets in at least two questionnaires).

*Place of living*

Rural vs. urban living at birth was based on the following questions: “Where did the mother live during pregnancy/year before pregnancy?” (reported at birth in ABIS) and “What type of house do you live in at 15-week pregnancy?” (reported at 15^th^ week of pregnancy in MoBa). Rural living was defined as living “alone in the countryside”, “a village up to 500 residents” (ABIS) and as living on “a farm” (MoBa). Urban living was in the ABIS cohort defined as living in “a city with 500-3000 residents” or “in a bigger city/town” and in the MoBa cohort as any other response alternative than farm.

*Older siblings*

We analyzed having an older sibling at birth (vs. no sibling), sibling order at birth (0, 1, 2, >3 older siblings), and per additional number of siblings. Similar categorization has previously been used.^2, 8-10^ The number of siblings was defined as siblings living at home at birth (ABIS) and based on parity data linked from the Medical Birth Registry of Norway^11^ (MoBa).

*Daycare attendance*

We examined daycare attendance by age 12 months (yes vs. no) and 36 months (yes vs. no). Similar to previous literature,^9, 10^ we defined daycare attendance as exposure to other children outside of the home using the response alternatives listed in Supplementary Table 2. Questionnaires were administered by 12 and 30-36 months (ABIS), and by 18 and 36 months (MoBa).

*Household crowding*

Household crowding (i.e., square meter (sqm) of living area per person) was based on the reported floor area and the number of persons living at home at birth in ABIS. In MoBa, a questionnaire administered at 15^th^ week of pregnancy was used to collect information about the number of persons living at home and information on floor area was reported in a questionnaire by the child’s age of 18 months. Motivated by Norwegian standards for household density,^12^ we defined household crowding as <25 sqm living area/person and categorized accordingly: <25, 25-50 and >50 sqm/person.

*Early-life drinking water*

The type of drinking water (public vs. private water) was assessed using questionnaire data collected at age 12 months (ABIS), and 18 months (MoBa).

*Infant bed-sharing (MoBa)*

In the MoBa cohort, we retrieved data on infant bed-sharing from the 6-month questionnaire: “Does your child share a bed with his/her mother/father (at least half the night)?” Response options were no, sometimes and often. Timing of bed-sharing was further specified as at birth, age two, four, and six months. Motivated by other studies,^2, 3^ we defined bed-sharing as reported “often” at minimum two timepoints by age six months of age (e.g., reported “often” at birth and at age two months). Data on bed-sharing were unavailable in the ABIS cohort.

## **Supplementary Table 2**. Harmonization of hygiene-related exposure variables in the ABIS and MoBa cohorts

| **Exposure** | **Description** | **ABIS** | **MoBa** |
| --- | --- | --- | --- |
| Place of living at birth | Place of living at birth  1. Rural =  - Alone on the countryside or Village up to 500 residents (ABIS)  - Farm (MoBa)  2. Urban =  - City with 500-3000 residents / in a bigger city/town (ABIS)  - other response option than Farm (MoBa) | At birth (Q32) Where did the mother live during pregnancy/year before pregnancy?  1. Alone on the countryside  2. Village up to 500 residents  3. City with 500-3000 residents  4. In a bigger city/town | 15-week pregnancy (Q81) What type of house do you live in?  1. Detached house  2. Farm  3. Semi detached  4. Four-flat house  5. Maisonette  6. Terraced flat  7. Basement flat  8. Apartment building  9. Townhouse/tenement  10. Which floor?  11. Other |
| Having pets at birth | Having pets at home at birth  1. No =  - Not having any pet at home (ABIS)  - Not having any pet at home or only having pet at childminder’s (MoBa)  2. Yes =  - Having any pet at home  (ABIS/MoBa) | At birth (Q11)  Did you have any indoor pets?  1. No  2. Dog  3. Cat  4. Bird  5. Guinea pig  6. Hamster  7. Exotic animal  8. Other, if yes which? | 6 months (Q75)  (Are there any pets in the child’s home?) If yes, which types?  1. Dog  2. Cat  3. Guinea pig, rabbit, mouse, rat, etc.  4. Budgie, other type of bird  5. Other type of animal |
| Duration of having pets from birth to 36 mo | Having pets at home at any timepoint the first three years  1. 0 year - Not reporting to have pets in any questionnaire  2. 1 year - Reporting to have pets at least in one questionnaire  3. >2 years - Reporting to have pets in two or more questionnaires | At birth (Q11)  12 months (Q29)  30–36 months(Q56)  Did you have any indoor pets?  1. No  2. Dog  3. Cat  4. Bird  5. Guinea pig  6. Hamster  7. Exotic animal  8. Other, if yes which? | 6 months (Q75)  (Are there any pets in the child’s home?) If yes, which types?  1. Dog  2. Cat  3. Guinea pig, rabbit, mouse, rat, etc.  4. Budgie, other type of bird  5. Other type of animal  18 months (Q53)  Are there pets where your child lives or at the childminder’s?  1. No  2. Yes, at home  3. Yes, at childminder’s  18 months (Q54)  (Yes, we have pets at home?) If yes, which types?  1. Dog  2. Cat  3. Guinea pig, rabbit, mouse, rat, etc.  4. Budgie, other type of bird  5. Other type of animal |
| Type of pet at birth | Type of pets at birth  1. Dog =  - Dog as pets at home  2. Cat - Cat as pets at home  3. Other/more than one pet  - Other type of pet at home  - If more than one pet at home was reported (e.g., cat and bird).  4. No pet - Not reporting any type of animal at home | At birth (Q11)  Did you have any indoor pets?  1. No  2. Dog  3. Cat  4. Bird  5. Guinea pig  6. Hamster  7. Exotic animal  8. Other, if yes which? | 6 months (Q75)  If yes, which types?  1. Dog  2. Cat  3. Guinea pig, rabbit, mouse, rat, etc.  4. Budgie, other type of bird  5. Other type of animal |
| Older siblings at birth | Number of siblings at birth  0. no = No sibling  1. yes = >1 sibling | At birth (Q4a)  (Does the child have any sibling?) If yes, how many?  1. 1  2. 2  3. 3  4. 4  5. 5  6. 6  7. 7  8. 8  9. 9 or more | Parity from the Medical Birth Registry of Norway ^11^ |
| Sibling order at birth | Number of older siblings at birth  0. No sibling  1. 1 sibling  2. 2 siblings  3. >3 sibling | At birth (Q4a)  (Does the child have any sibling?) If yes, how many?  1. 1  2. 2  3. 3  4. 4  5. 5  6. 6  7. 7  8. 8  9. 9 or more | Parity from the Medical Birth Registry of Norway^11^ |
| Daycare by 12 months | Use of daycare outside home at 1 year  0. No =  - Not attending any type of daycare (ABIS)  - Cared at home with family member or cared at home with an unqualified childminder (MoBa)  1. Yes =  - Childminder/daycare (ABIS)  - At childminder/In a family day nursery/In a day nursery (MoBa) | 12 months (Q31a, Q32a)  Is the child cared for/have the child been cared for at childminder’s/daycare?  1. Yes  2. No | 18 months (Q41)  Where has your child been cared for during the day? Enter a cross for the various age groups. (at home with his/her father or mother/at home with unqualified childminder, at childminder’s/in a day nursery)  1. 0-6 months  2. 7-9 months  3.10-12 months |
| Daycare by 36 months | Use of daycare outside home at 3 years  0. No =  - Not attending any type of daycare (ABIS)  - Cared at home with family member or cared at home with an unqualified childminder (MoBa)  1. Yes =  - Childminder/daycare (ABIS)  - At a childminder’s/In a short term outdoor day nursery/In a day nursery (MoBa) | 30-36 months (Q60, Q62)  Is the child cared for/have the child been cared for at childminder’s/daycare?  1. Yes  2. No | 36 months (Q39)  How is your child cared for during the day at the moment?  1. At home with his/her mother  2. At home with his/her father  3. At home with an unqualified childminder  4. At a childminder’s  5. In a short term outdoor day nursery  6. In a day nursery |
| Degree household crowding at birth (sqm/ person) | Degree household crowding at birth (sqm/person)  1. <25  2. 25-50  3. >50  Household crowding was calculated by square meters divided with number of people living in household at birth, and thereafter divided into categories of <25, 25-50, >50- | At birth (Q5)  How many people live in the same house (newborn not included)?  1. 1  2. 2  3. 3  4. 4  5. 5  6. 6  7. 7  8. 8  9. 9 or more  At birth (Q7)  How many square meters? | 15 week pregnant (Q73) How many people including you live in your home?  1. Number of people over 18 years  2. Number of people between 12 - 18 years  3. Number of people between 6 - 11 years  4. Number of people under 6 years  18 months (Q47)  Roughly how many square meters is the living area where you currently live? |
| Early-life drinking water | Type of water early in life?  1. Private water=  - Own well (ABIS)  - Water from your own water supply (e.g., own well) (MoBa)  2. Public water=  - Municipal water (ABIS)  - Water from a public or private water company (MoBa)  Don’t know was coded as missing. | 12 months (Q28)  Where did the water that the child mostly drank come from?  1. Own well  2. Municipal water  3. Don’t know | 18 months (Q51) What type of drinking water do you have where you live?  1. Water from a public or private water company  2. Water from your own water supply (e.g. own well)  3. Donʼt know |
| Bed-sharing at 0-6 months | Sharing bed with other family member at least half of the night the first 6 months of life?  1. Yes= sharing bed often > 2 timepoints  2. No = no/sometimes bed-sharing | *Data were not available* | 6 months (Q44)  Does your child share a bed with his/her mother/father (at least half the night)? (no/sometimes/often)  1. After birth  2. At 2 months  3. At 4 months  4. At 6 months |

ABIS, All Babies in Southeast Sweden study; MoBa, The Norwegian Mother, Father and Child Cohort Study.

## **Supplementary Table 3.** Definition of inflammatory bowel disease^a^

| **Disease** | **ICD-10 (1997-)** | **Description** |
| --- | --- | --- |
| Inflammatory bowel disease | K52.3, CD + UC or mixed diagnosis of CD, UC and IBD-U | IBD diagnosis required >2 inpatient or non-primary outpatient care visits. Patients who shifted between subtypes of IBD, but only had one diagnosis the last five years were classified accordingly to their most recent diagnosis. Patients with several codes of CD, UC or IBD-U during follow-up was classified as (“any, non-specific”) IBD. |
| Crohn’s disease | K50 |  |
| Ulcerative colitis | K51 |  |
| IBD-unclassified | K52.3 |  |

^a^Excerpt of IBD definition from Everhov et. al.^13^ CD, Crohn’s disease, IBD, inflammatory bowel disease; IBD-U, inflammatory bowel disease unclassified; ICD-10, International Classification of Diseases, Tenth Revision code.

## **Supplementary Table 4.** Prevalence and incidence with 95% CI for inflammatory bowel disease, Crohn’s disease, and ulcerative colitis in the ABIS and MoBa cohorts

|  | **N event** | **Person-years of follow-up** | **Incidence rate per 100,000 PYR** | **Prevalence at end of**  **follow-up^a^** |
| --- | --- | --- | --- | --- |
| ABIS |  |  |  |  |
| IBD | 113 | 360,655 | 31.33 (25.82, 37.67) | 0.70% (0.57, 0.84) |
| CD | 40 | 359,401 | 11.13 (7.95, 15.16) | 0.25% (0.18, 0.34) |
| UC | 57 | 359,713 | 15.85 (12.00, 20.53) | 0.35% (0.27, 0.45) |
| IBD-U | 16 | 361,188 | 4.43 (2.53, 7.19) | 0.10% (0.06, 0.16) |
| MoBa |  |  |  |  |
| IBD | 338 | 1,663,644 | 20.32 (18.21, 22.60) | 0.33% (0.30, 0.37) |
| CD | 142 | 1,661,146 | 8.55 (7.20, 10.08) | 0.14% (0.12, 0.17) |
| UC | 95 | 1,664,597 | 5.72 (4.63, 6.99) | 0.09% (0.08, 0.11) |
| IBD-U | 101 | 1,664,579 | 6.07 (4.94, 7.37) | 0.10% (0.08, 0.12) |

^a^December 31, 2020 (ABIS) and December 31, 2021 (MoBa). ABIS, All Babies in Southeast Sweden study; CD, Crohn’s disease; CI, confidence interval; IBD, inflammatory bowel disease; IBD-U, IBD-unclassified (cases with where subtype could not be distinguished from CD or UC) ; MoBa, The Norwegian Mother, Father and Child Cohort Study; PYR, person-year; UC, ulcerative colitis.

## **Supplementary Table 5.** Characteristics of included and excluded participants in the ABIS and MoBa cohorts

|  | **ABIS** | | | **MoBa** | | | |
| --- | --- | --- | --- | --- | --- | --- | --- |
|  | **Included**  **at birth**  **(n=16,223)** | **Excluded**  **at 12 months (n=5,360)** | **Excluded**  **at 36 months (n=7,546)** | **Included**  **at 15 GW**  **(n=101,270)** | **Excluded**  **at 6 months**  **(n=26,041)** | **Excluded  at 18 months (n=12,465)** | **Excluded**  **at 36 months (n=14,740)** |
| Incidence rate per 100,000 PYR (95% CI) | | | | | | | |
| IBD | 31.39 (25.87, 37.74) | 32.00 (22.64, 43.92) | 29.29 (21.67, 38.72) | 20.32 (18.21, 22.60) | 21.57 (18.09-25.53) | 20.74 (17.74-24.09) | 19.78 (17.20-22.65) |
| CD | 11.08 (7.92, 15.09) | 9.23 (4.61, 16.51) | 11.92 (7.28. 18.40) | 8.53 (7.19, 10.06) | 7.50 (5.51-9.98) | 7.76 (5.97-9.90) | 7.53 (5.97-9.37) |
| UC | 15.80 (11.96, 20.47) | 18.47 (11.57, 27.96) | 14.30 (9.16, 21.28) | 5.71 (4.62, 6.98) | 5.91 (4.16-8.14) | 5.82 (4.29-7.71) | 5.84 (4.48-7.48) |
| Maternal smoking, n % | | | | | | | |
| Yes | 1760 (11.1) | 775 (14.5) | 1021 (13.5) | 9597 (9.5) | 3552 (13.6) | 1381 (11.1) | 1353 (9.2) |
| Missing | 380 (2.3) | 143 (2.7) | 188 (2.5) | 1587 (1.6) | 1207 (4.6) | 82 (0.7) | 62 (0.4) |
| Maternal age at delivery (years), n %^a^ | | | | | | | |
| <17-24 | 2548 (15.6) | 1026 (18.0) | 1401 (18.6) | 11,071 (10.9) | 3684 (14.2) | 1693 (13.6) | 1688 (11.5) |
| 25-34 | 11,440 (70.5) | 3631 (67.8) | 5139 (68.1) | 72,431 (71.5) | 17,953 (69.0) | 8680 (69,6) | 10,556 (71.7) |
| 35-44 | 1972 (12.1) | 631 (11.8) | 882 (11.6) | 17,714 (17.5) | 4394 (16.9) | 2081 (16.7) | 2491 (16.9) |
| Missing | 263 (1.6) | 72 (1.3) | 124 (1.6) | 54 (0.1) | 10 (0.0) | 11 (0.1) | 5 (0.0) |

^a^Maternal age <15 years and >44 years at delivery were considered implausible and changed to missing. ABIS, All Babies in Southeast Sweden study; CD, Crohn’s disease; IBD, inflammatory bowel disease; MoBa, The Norwegian Mother, Father and Child Cohort Study; PYR, person-years; UC, ulcerative colitis.

## **Supplementary Table 6.** Pooled hazard ratios for the risk of inflammatory bowel disease, Crohn’s disease and ulcerative colitis adjusted for perinatal characteristics^a^

| **Characteristics** | **IBD**  **HR (95% CI)** | **Crohn’s disease**  **HR (95% CI)** | **Ulcerative colitis**  **HR (95%CI)** |
| --- | --- | --- | --- |
| Place of living at birth |  |  |  |
| Urban | Ref | Ref | Ref |
| Rural | 1.26 (0.86, 1.85) | 1.52 (0.89, 2.62) | 1.02 (0.51, 2.06) |
| Having pets at birth |  |  |  |
| No | Ref | Ref | Ref |
| Yes | 1.11 (0.86, 1.42) | 1.11 (0.76, 1.61) | 1.05 (0.67, 1.63) |
| Duration of having pets from birth to 36 mo | | |  |
| 0 year | Ref | Ref | Ref |
| 1 year | 1.08 (0.74, 1.58) | 0.97 (0.53, 1.78) | 1.17 (0.61, 2.25) |
| >2 years | 1.03 (0.76, 1.40) | 1.09 (0.69, 1.72) | 0.95 (0.54, 1.66) |
| Type of pet at birth |  |  |  |
| No pet | Ref | Ref | Ref |
| Cat | 1.09 (0.66, 1.79) | 1.09 (0.48, 2.43) | 1.05 (0.57, 1.93) |
| Dog | 1.25 (0.74, 2.10) | 1.07 (0.43, 2.67) | 1.26 (0.66, 2.41) |
| Other | 0.91 (0.52, 1.61) | 0.99 (0.42, 2.37) | 0.82 (0.37, 1.82) |
| Older siblings at birth |  |  |  |
| 0 | Ref | Ref | Ref |
| >1 | 1.20 (0.94, 1.53) | 1.04 (0.54, 2.00) | 1.34 (0.67, 2.69) |
| Sibling order at birth |  |  |  |
| 0 | Ref | Ref | Ref |
| 1 | 1.16 (0.90, 1.50) | 0.98 (0.47, 2.06) | 1.14 (0.62, 2.09) |
| 2 | 1.26 (0.89, 1.77) | 1.10 (0.49, 2.48) | 1.67 (0.83, 3.34) |
| >3 | 1.53 (0.92, 2.54) | 1.04 (0.36, 3.01) | 1.73 (0.63, 4.75) |
| Per additional older sibling |  |  |  |
|  | 1.14 (0.99, 1.31) | 1.06 (0.86, 1.30) | 1.25 (0.92, 1.69) |
| Daycare by age 12 months |  |  |  |
| No | Ref | Ref | Ref |
| Yes | 1.20 (0.60, 2.38) | 1.58 (0.90, 2.78) | - |
| Daycare by age 36 months | |  |  |
| No | Ref | Ref | Ref |
| Yes | 0.73 (0.51, 1.01) | 0.57 (0.35, 0.93) | 1.09 (0.51, 2.34) |
| Degree household crowding at birth (sqm/person) | | |  |
| >50 | Ref | Ref | Ref |
| 25-50 | 0.77 (0.50, 1.16) | 0.70 (0.47, 1.04) | 1.07 (0.51, 2.23) |
| <25 | 1.20 (0.63, 2.29) | 1.04 (0.44, 2.44) | 1.61 (0.45, 5.74) |
| Early-life drinking water |  |  |  |
| Public water | Ref | Ref | Ref |
| Private water | 1.15 (0.42, 3.13) | 1.17 (0.61, 2.26) | 0.80 (0.18. 3.51) |

^a^Adjusted model 2 (secondary model): adjusted for sex, parental inflammatory bowel disease, parental origin, parental education level, maternal comorbidities (type 1 diabetes, autoimmune thyroid disease, rheumatoid arthritis), delivery mode, maternal smoking, maternal age, birth weight, gestational age (weeks), full breastfeeding. CI, confidence interval; HR, hazard ratio; Ref, reference category.

## **Supplementary Table 7.** Hygiene-related exposures of early-life and later risk of inflammatory bowel disease in the ABIS and MoBa cohorts

|  | **N** | **Events n** | **Incidence per 100,000 PYR** | **Unadjusted HR (95% CI)** | **Adjusted^a^ HR (95% CI)** | **Adjusted^b^**  **HR (95% CI)** |
| --- | --- | --- | --- | --- | --- | --- |
| Place of living at birth |  |  |  |  |  |  |
| ABIS | 16,223 | 113 |  |  |  |  |
| Urban | 11,962 (73.7) | 87 (77.0) | 32.7 (26.2, 40.3) | Ref | Ref | Ref |
| Rural | 3600 (22.2) | 23 (20.3) | 28.8 (18.2, 43.1) | 0.88 (0.56, 1.39) | 0.87 (0.54, 1.40) | 1.26 (0.69, 2.30) |
| Missing | 661 (4.1) | 3 (2.7) |  |  |  |  |
| MoBa | 101,270 | 338 |  |  |  |  |
| Urban | 95,448 (94.3) | 315 (93.2) | 20.1 (18.0, 22.5) | Ref | Ref | Ref |
| Rural | 4403 (4.3) | 19 (5.6) | 25.8 (15.5, 40.3) | 1.24 (0.78, 1.97) | 1.22 (0.76, 1.97) | 1.26 (0.77, 2.07) |
| Missing | 1419 (1.4) | 4 (1.2) |  |  |  |  |
| Having pets at birth |  |  |  |  |  |  |
| ABIS | 16,223 | 113 |  |  |  |  |
| No | 9240 (57.0) | 64 (56.6) | 31.2 (24.0, 39.8) | Ref | Ref | Ref |
| Yes | 6983 (43.0) | 49 (43.4) | 31.6 (23.4, 41.7) | 1.01 (0.70, 1.47) | 0.98 (0.67, 1.44) | 1.03 (0.61, 1.76) |
| Missing | 0 | 0 |  |  |  |  |
| MoBa | 75,229 | 239 |  |  |  |  |
| No | 53,279 (70.8) | 160 (66.9) | 18.7 (15.9, 21.9) | Ref | Ref | Ref |
| Yes | 21,523 (28.6) | 76 (31.8) | 21.8 (17.2, 27.3) | 1.15 (0.87, 1.51) | 1.15 (0.87, 1.52) | 1.13 (0.85, 1.50) |
| Missing | 427 (0.6) | 3 (1.3) |  |  |  |  |
| Duration of having pets from birth to 36 mo | | |  |  |  |  |
| ABIS | 8667 | 64 |  |  |  |  |
| 0 year | 4143 (47.7) | 28 (43.8) | 30.4 (20.2, 43.9) | Ref | Ref | Ref |
| 1 year | 1096 (12.6) | 6 (9.4) | 24.6 (9.0, 53.5) | 0.81 (0.33, 1.95) | 0.89 (0.37, 2.15) | 0.77 (0.23, 2.65) |
| >2 years | 3438 (39.6) | 30 (46.9) | 39.2 (26.5, 56.0) | 1.29 (0.77, 2.16) | 1.41 (0.83, 2.39) | 1.21 (0.59, 2.47) |
| Missing | 0 | 0 |  |  |  |  |
| MoBa | 62,764 | 203 |  |  |  |  |
| 0 year | 39,113 (62.3) | 121 (59.6) | 19.3 (16.1, 23.1) | Ref | Ref | Ref |
| 1 year | 8357 (13.3) | 31 (15.3) | 22.6 (15.3, 32.1) | 1.11 (0.75, 1.65) | 1.12 (0.75, 1.67) | 1.12 (0.75, 1.67) |
| >2 years | 15,294 (24.4) | 51 (25.1) | 20.6 (15.3, 27.0) | 1.03 (0.75, 1.44) | 1.02 (0.73, 1.42) | 0.99 (0.71, 1.39) |
| Missing | 0 | 0 |  |  |  |  |
| Type of pet at birth |  |  |  |  |  |  |
| ABIS | 16,223 | 113 |  |  |  |  |
| No pets | 9240 (57.0) | 64 (56.7) | 31.2 (24.0, 39.8) | Ref | Ref | Ref |
| Cat | 2804 (17.3) | 20 (17.7) | 32.1 (19.6, 49.5) | 1.03 (0.62, 1.70) | 1.02 (0.61, 1.70) | 0.80 (0.37, 1.76) |
| Dog | 1826 (11.3) | 11 (9.7) | 27.1 (13.5, 48.5) | 0.87 (0.46, 1.65) | 0.86 (0.45, 1.63) | 0.93 (0.39, 2.23) |
| Other | 2353 (14.5) | 18 (15.9) | 34.4 (20.4, 54.4) | 1.10 (0.65, 1.86) | 1.04 (0.61, 1.79) | 1.41 (0.70, 2.83) |
| Missing | 0 | 0 |  |  |  |  |
| MoBa | 75,229 | 239 |  |  |  |  |
| No pets | 53,279 (70.8) | 160 (66.9) | 18.7 (15.9, 21.9) | Ref | Ref | Ref |
| Cat | 9356 (12.4) | 36 (15.1) | 23.9 (16.7, 33.1) | 1.26 (0.88, 1.81) | 1.25 (0.86, 1.81) | 1.24 (0.86, 1.80) |
| Dog | 6702 (8.9) | 29 (12.1) | 26.8 (18.0, 38.5) | 1.42 (0.95, 2.10) | 1.44 (0.97, 2.14) | 1.40 (0.93, 2.09) |
| Other | 5465 (7.3) | 11 (4.6) | 12.3 (6.1, 22.0) | 0.63 (0.34, 1.17) | 0.64 (0.34, 1.18) | 0.63 (0.34, 1.16) |
| Missing | 427 (0.6) | 3 (1.3) |  |  |  |  |
| Older siblings at birth |  |  |  |  |  |  |
| ABIS | 16,223 | 113 |  |  |  |  |
| 0 | 5966 (36.8) | 40 (35.4) | 30.1 (21.5, 41.0) | Ref | Ref | Ref |
| >1 | 9832 (60.6) | 71 (62.8) | 32.5 (25.4, 41.0) | 1.08 (0.73, 1.59) | 1.07 (0.72, 1.57) | 1.07 (0.60, 1.93) |
| Missing | 425 (2.6) | 2 (1.8) |  |  |  |  |
| MoBa | 101,270 | 338 |  |  |  |  |
| 0 | 45,421 (44.9) | 136 (40.2) | 18.4 (15.4, 21.8) | Ref | Ref | Ref |
| >1 | 55,849 (55.1) | 202 (59.8) | 21.9 (18.9, 25.1) | 1.15 (0.93, 1.43) | 1.20 (0.96, 1.51) | 1.23 (0.94, 1.60) |
| Missing | 0 | 0 |  |  |  |  |
| Sibling order at birth |  |  |  |  |  |  |
| ABIS | 16,223 | 113 |  |  |  |  |
| 0 | 5966 (36.8) | 40 (35.4) | 30.1 (21.5, 41.0) | Ref | Ref | Ref |
| 1 | 5983 (36.9) | 39 (34.5) | 29.3 (20.8, 40.1) | 0.97 (0.63, 1.51) | 0.97 (0.63, 1.52) | 0.97 (0.51, 1.84) |
| 2 | 2618 (16.1) | 18 (15.9) | 31.0 (18.4, 48.9) | 1.03 (0.59, 1.79) | 1.03 (0.59, 1.80) | 1.12 (0.48, 2.59) |
| >3 | 1231 (7.6) | 14 (12.4) | 51.2 (28.0, 85.8) | 1.70 (0.92, 3.12) | 1.59 (0.85, 2.98) | 2.21 (0.83, 5.84) |
| Missing | 425 (2.6) | 2 (1.8) |  |  |  |  |
| MoBa | 101,270 | 338 |  |  |  |  |
| 0 | 45,421 (44.9) | 136 (40.2) | 18.4 (15.4, 21.8) | Ref | Ref | Ref |
| 1 | 36,107 (35.7) | 123 (36.4) | 20.7 (17.2, 24.7) | 1.10 (0.86, 1.40) | 1.16 (0.90, 1.49) | 1.20 (0.91, 1.60) |
| 2 | 15,391 (15.2) | 61 (18.0) | 23.8 (18.2, 30.5) | 1.24 (0.91, 1.67) | 1.25 (0.91, 1.72) | 1.29 (0.89, 1.87) |
| >3 | 4351 (4.3) | 18 (5.3) | 24.7 (14.6, 39.0) | 1.27 (0.78, 2.08) | 1.39 (0.84, 2.29) | 1.33 (0.73, 2.42) |
| Missing | 0 | 0 |  |  |  |  |
| Per additional older sibling | |  |  |  |  |  |
| ABIS | 15,798 | 111 | - | 1.13 (0.93, 1.38) | 1.11 (0.91, 1.36) | 1.21 (0.88, 1.67) |
| MoBa | 101,270 | 338 | - | 1.10 (0.97, 1.24) | 1.12 (0.99, 1.27) | 1.12 (0.96, 1.31) |
| Daycare by age 12 months |  |  |  |  |  |  |
| ABIS | 10,863 | 75 |  |  |  |  |
| No | 9172 (84.4) | 66 (88.0) | 32.4 (25.0, 41.2) | Ref | Ref | Ref |
| Yes | 628 (5.8) | 4 (5.3) | 28.5 (7.8, 73.0) | 0.88 (0.32, 2.41) | 0.66 (0.21, 2.10) | 0.59 (0.14, 2.44) |
| Missing | 1063 (9.8) | 5 (6.7) |  |  |  |  |
| MoBa | 62,764 | 203 |  |  |  |  |
| No | 56,985 (90.8) | 178 (87.7) | 19.3 (16.6, 22.4) | Ref | Ref | Ref |
| Yes | 5779 (9.2) | 25 (12.3) | 27.6 (17.9, 40.7) | 1.51 (0.99, 2.30) | 1.40 (0.91, 2.17) | 1.43 (0.92, 2.21) |
| Missing | 0 | 0 |  |  |  |  |
| Daycare by age 36 months |  |  |  |  |  |  |
| ABIS | 8,677 | 64 |  |  |  |  |
| No | 897 (10.3) | 8 (12.5) | 40.1 (17.3, 78.9) | Ref | Ref | Ref |
| Yes | 7780 (89.7) | 56 (87.5) | 32.4 (24.4, 42.0) | 0.81 (0.39, 1.70) | 0.81 (0.38, 1.70) | 0.69 (0.27, 1.79) |
| Missing | 0 | 0 |  |  |  |  |
| MoBa | 48,024 | 164 |  |  |  |  |
| No | 12,492 (26.0) | 41 (25.0) | 22.5 (16.2, 30.5) | Ref | Ref | Ref |
| Yes | 35,532 (74.0) | 123 (75.0) | 20.8 (17.3, 24.8) | 0.72 (0.50, 1.03) | 0.71 (0.49, 1.02) | 0.72 (0.50, 1.04) |
| Missing | 0 | 0 |  |  |  |  |
| Household crowding at birth (sqm/person) | |  |  |  |  |  |
| ABIS | 15,125 | 113 |  |  |  |  |
| >50 | 4501 (27.7) | 33 (29.2) | 33.0 (22.7, 46.4) | Ref | Ref | Ref |
| 25-50 | 9487 (58.5) | 62 (54.9) | 29.4 (22.5, 37.7) | 0.89 (0.58, 1.35) | 0.87 (0.57, 1.32) | 0.77 (0.42, 1.42) |
| <25 | 1137 (7.0) | 11 (9.7) | 43.6 (21.7, 77.9) | 1.32 (0.67, 2.61) | 1.10 (0.54, 2.27) | 2.10 (0.89, 4.98) |
| Missing | 1098 (6.8) | 7 (6.2) |  |  |  |  |
| MoBa | 62,764 | 203 |  |  |  |  |
| >50 | 31,651 (50.4) | 118 (58.1) | 23.1 (19.1, 27.7) | Ref | Ref | Ref |
| 25-50 | 26,038 (41.5) | 73 (36.0) | 17.4 (13.7, 21.9) | 0.76 (0.56, 1.01) | 0.75 (0.56, 1.01) | 0.76 (0.57, 1.03) |
| <25 | 2494 (4.0) | 7 (3.4) | 17.5 (7.0, 31.6) | 0.77 (0.36, 1.64) | 0.74 (0.35, 1.61) | 0.76 (0.35, 1.64) |
| Missing | 2581 (4.1) | 5 (2.5) |  |  |  |  |
| Early-life drinking water |  |  |  |  |  |  |
| ABIS | 10,863 | 75 |  |  |  |  |
| Public water | 8650 (79.6) | 54 (72.0) | 28.1 (21.1, 36.6) | Ref | Ref | Ref |
| Private water | 2213 (20.4) | 21 (28.0) | 42.7 (26.4, 65.2) | 1.52 (0.92, 2.52) | 1.51 (0.90, 2.54) | 1.93 (1.06, 3.52) |
| Missing | 0 | 0 |  |  |  |  |
| MoBa | 62,764 | 203 |  |  |  |  |
| Public water | 56,838 (90.6) | 188 (92.6) | 20.6 (17.7, 23.7) | Ref | Ref | Ref |
| Private water | 5242 (8.4) | 14 (6.9) | 16.2 (8.9, 27.2) | 0.76 (0.44, 1.30) | 0.69 (0.39, 1.21) | 0.69 (0.39, 1.22) |
| Missing | 684 (1.1) | 1 (0.5) |  |  |  |  |
| Bed-sharing 0-6 months |  |  |  |  |  |  |
| MoBa | 75,229 | 239 |  |  |  |  |
| No | 57,999 (77.1) | 169 (70.7) | 18.1 (15.5, 21.1) | Ref | Ref | Ref |
| Yes | 16,719 (22.2) | 66 (27.6) | 24.5 (19.0, 31.2) | 1.35 (1.02, 1.80) | 1.35 (1.01, 1.79) | 1.32 (0.99, 1.77) |
| Missing | 511 (0.7) | 4 (1.7) |  |  |  |  |

^a^Adjusted model 1 (primary model): adjusted for sex, parental inflammatory bowel disease, parental origin, parental education level, maternal comorbidities (type 1 diabetes, autoimmune thyroid disease, rheumatoid arthritis). ^b^Adjusted model 2 (secondary model): also adjusted for delivery mode, maternal smoking, maternal age, birth weight, gestational age (weeks), full breastfeeding. ABIS, All Babies in Southeast Sweden study; CI, confidence interval; HR, hazard ratio; MoBa, The Norwegian Mother, Father and Child Cohort Study; PYR, person-year; Ref, reference category.

## **Supplementary Table 8.** Hygiene-related exposures of early-life and later risk of Crohn’s disease in the ABIS and MoBa cohorts

|  | **N** | **Events n** | **Incidence per 100,000 PYR** | **Unadjusted HR (95% CI)** | **Adjusted^a^ HR (95% CI)** | **Adjusted^b^**  **HR (95% CI)** |
| --- | --- | --- | --- | --- | --- | --- |
| Place of living at birth |  |  |  |  |  |  |
| ABIS | 16,223 | 40 |  |  |  |  |
| Urban | 11,905 (73.4) | 30 (75.0) | 11.3 (7.6, 16.2) | Ref | Ref | Ref |
| Rural | 3587 (22.1) | 10 (15.0) | 12.5 (6.0, 23.1) | 11.1 (0.54, 2.27) | 1.04 (0.49, 2.21) | 1.51 (0.62, 3.66) |
| Missing | 731 (4.5) | 0 (0.0) |  |  |  |  |
| MoBa | 101,270 | 142 |  |  |  |  |
| Urban | 95,265 (94.1) | 132 (3.0) | 8.4 (7.1, 10.0) | Ref | Ref | Ref |
| Rural | 4393 (4.3) | 9 (6.3) | 12.3 (5.6, 23.3) | 1.41 (0.72, 2.78) | 1.46 (0.74, 2.88) | 1.54 (0.77, 3.04) |
| Missing | 1612 (1.6) | 1 (0.7) |  |  |  |  |
| Having pets at birth |  |  |  |  |  |  |
| ABIS | 16,223 | 40 |  |  |  |  |
| No | 9201 (56.7) | 25 (62.5) | 12.2 (7.9, 18.0) | Ref | Ref | Ref |
| Yes | 6949 (42.8) | 15 (37.5) | 9.7 (5.4, 16.0) | 0.79 (0.42, 1.51) | 0.72 (0.37, 1.41) | 0.83 (0.37, 1.87) |
| Missing | 73 (1.1) | 0 (0.0) |  |  |  |  |
| MoBa | 75,229 | 108 |  |  |  |  |
| No | 53,190 (70.7) | 71 (65.7) | 8.3 (6.5, 10.5) | Ref | Ref | Ref |
| Yes | 21,483 (28.6) | 36 (33.3) | 10.4 (7.3, 14.3) | 1.23 (0.82, 1.84) | 1.24 (0.82, 1.88) | 1.19 (0.79, 1.82) |
| Missing | 556 (0.7) | 1 (0.9) |  |  |  |  |
| Duration of having pets from birth to 36 mo | | |  |  |  |  |
| ABIS | 8677 | 20 |  |  |  |  |
| 0 year | 4127 (47.6) | 12 (60.0) | 13.1 (6.7, 22.8) | Ref | Ref | Ref |
| 1 year | 1091 (12.6) | 1 (5.0) | 4.1 (0.1, 22.9) | 0.31 (0.04, 2.42) | 0.34 (0.04, 2.61) | 0.57 (0.07, 4.62) |
| >2 years | 3415 (39.4) | 7 (35.0) | 9.2 (3.7, 19.0) | 0.70 (0.28, 1.79) | 0.74 (0.29, 1.91) | 0.89 (0.28, 2.80) |
| Missing | 44 (0.5) | 0 (0.0) |  |  |  |  |
| MoBa | 62,764 | 91 |  |  |  |  |
| 0 year | 39,045 (62.2) | 53 (58.2) | 8.5 (6.4, 11.1) | Ref | Ref | Ref |
| 1 year | 8338 (13.3) | 12 (13.2) | 8.7 (4.5, 15.3) | 1.00 (0.54, 1.88) | 1.03 (0.54, 1.93) | 1.02 (0.54, 1.92) |
| >2 years | 15,269 (24.3) | 26 (28.6) | 10.5 (6.9, 15.4) | 1.22 (0.76, 1.95) | 1.19 (0.73, 1.94) | 1.14 (0.69, 1.86) |
| Missing | 112 (0.2) | 0 |  |  |  |  |
| Type of pet at birth |  |  |  |  |  |  |
| ABIS | 16,223 | 40 |  |  |  |  |
| No pets | 9201 (56.7) | 25 (62.5) | 12.2 (7.9, 18.0) | Ref | Ref | Ref |
| Cat | 2790 (17.2) | 6 (15.0) | 9.7 (3.5, 21.0) | 0.79 (0.32, 1.93) | 0.78 (0.32, 1.90) | 0.62 (0.18, 2.14) |
| Dog | 1816 (11.2) | 1 (2.5) | 2.5 (0.1, 13.8) | 0.20 (0.03, 1.50) | 0.20 (0.03, 1.46) | 0.32 (0.04, 2.43) |
| Other | 2343 (14.4) | 8 (2.0) | 15.3 (6.6, 30.2) | 1.26 (0.57, 2.79) | 1.08 (0.46, 2.52) | 1.52 (0.58, 3.96) |
| Missing | 73 (0.4) | 0 (0.0) |  |  |  |  |
| MoBa | 75,229 | 108 |  |  |  |  |
| No pets | 53,190 (70.7) | 71 (65.7) | 8.3 (6.5, 10.5) | Ref | Ref | Ref |
| Cat | 9338 (12.4) | 18 (16.7) | 12.0 (7.1, 18.9) | 1.43 (0.85, 2.40) | 1.40 (0.82, 2.39) | 1.38 (0.81, 2.36) |
| Dog | 6686 (8.9) | 13 (12.0) | 12.0 (6.4, 20.6) | 1.44 (0.79, 2.59) | 1.50 (0.83, 2.72) | 1.38 (0.75, 2.56) |
| Other | 5459 (7.3) | 5 (4.6) | 5.6 (1.8, 13.0) | 0.65 (0.26, 1.62) | 0.67 (0.27, 1.66) | 0.66 (0.27, 1.65) |
| Missing | 556 (0.7) | 1 (0.9) |  |  |  |  |
| Older siblings at birth |  |  |  |  |  |  |
| ABIS | 16,223 | 40 |  |  |  |  |
| 0 (none) | 5945 (36.6) | 19 (47.5) | 14.4 (8.6, 22.4) | Ref | Ref | Ref |
| >1 | 9782 (60.3) | 21 (52.5) | 9.7 (6.0, 14.7) | 0.67 (0.36, 1.25) | 0.62 (0.33, 1.17) | 0.65 (0.28, 1.55) |
| Missing | 496 (3.1) | 0 |  |  |  |  |
| MoBa | 101,270 | 142 |  |  |  |  |
| 0 (none) | 45,338 (44.8) | 53 (37.3) | 7.2 (5.4, 9.4) | Ref | Ref | Ref |
| >1 | 55,736 (55.0) | 89 (62.7) | 9.6 (7.7, 11.9) | 1.31 (0.93, 1.84) | 1.31 (0.92, 1.86) | 1.32 (0.88, 1.99) |
| Missing | 196 (0.2) | 0 |  |  |  |  |
| Sibling order at birth |  |  |  |  |  |  |
| ABIS | 16,223 | 40 |  |  |  |  |
| 0 | 5945 (36.6) | 19 (47.5) | 14.4 (8.6, 22.4) | Ref | Ref | Ref |
| 1 | 5953 (36.6) | 9 (22.5) | 6.8 (3.1, 12.9) | 0.47 (0.21, 1.04) | 0.46 (0.21, 1.02) | 0.49 (0.17, 1.36) |
| 2 | 2606 (16.1) | 6 (15.0) | 10.4 (3.8, 22.6) | 0.72 (0.29, 1.81) | 0.71 (0.28, 1.78) | 0.79 (0.23, 2.72) |
| >3 | 1223 (7.5) | 6 (15.0) | 22.0 (8.1, 48.0) | 1.53 (0.61, 3.84) | 1.23 (0.46, 3.33) | 1.81 (0.48, 6.85) |
| Missing | 496 (3.1) | 0 |  |  |  |  |
| MoBa | 101,270 | 142 |  |  |  |  |
| 0 | 45,338 (44.8) | 53 (37.3) | 7.2 (5.4, 9.4) | Ref | Ref | Ref |
| 1 | 36,043 (35.6) | 59 (41.5) | 9.9 (7.6, 12.8) | 1.36 (0.94, 1.97) | 1.40 (0.95, 2.04) | 1.38 (0.90, 2.10) |
| 2 | 15,355 (15.2) | 25 (17.6) | 9.8 (6.3, 14.4) | 1.31 (0.82, 2.11) | 1.22 (0.74, 2.02) | 1.27 (0.72, 2.24) |
| >3 | 4338 (4.3) | 5 (3.5) | 6.9 (2.2, 16.0) | 0.92 (0.37, 2.30) | 0.95 (0.38, 2.40) | 0.64 (0.19, 2.16) |
| Missing | 196 (0.2) | 0 |  |  |  |  |
| Per additional older sibling | |  |  |  |  |  |
| ABIS | 15,727 | 40 | - | 1.04 (0.74, 1.45) | 0.97 (0.68, 1.37) | 1.11 (0.69, 1.79) |
| MoBa | 101,074 | 142 | - | 1.09 (0.90, 1.31) | 1.07 (0.89, 1.30) | 1.04 (0.82, 1.32) |
| Daycare by age 12 months |  |  |  |  |  |  |
| ABIS | 10,863 | 29 |  |  |  |  |
| No | 9132 (84.1) | 26 (89.7) | 12.8 (8.4, 18.7) | Ref | Ref | Ref |
| Yes | 627 (5.8) | 3 (10.3) | 21.4 (4.4, 62.6) | 1.68 (0.51, 5.55) | 1.12 (0.27, 4.74) | 1.33 (0.31, 5.74) |
| Missing | 1104 (10.2) | 0 |  |  |  |  |
| MoBa | 62,764 | 91 |  |  |  |  |
| No | 56,884 (90.6) | 77 (84.6) | 8.4 (6.6, 10.5) | Ref | Ref | Ref |
| Yes | 5768 (9.2) | 14 (15.4) | 15.5 (8.5, 26.0) | 1.91 (1.08, 3.38) | 1.66 (0.90, 3.05) | 1.63 (0.88, 3.00) |
| Missing | 112 (0.2) | 0 |  |  |  |  |
| Daycare by age 36 months |  |  |  |  |  |  |
| ABIS | 8,677 | 20 |  |  |  |  |
| No | 891 (10.3) | 2 (10.0) | 10.1 (1.2, 36.4) | Ref | Ref | Ref |
| Yes | 7742 (89.2) | 18 (90.0) | 10.4 (6.2, 16.5) | 1.04 (0.24, 4.46) | 1.05 (0.24, 4.53) | 0.66 (0.15, 2.98) |
| Missing | 44 (0.5) | 0 |  |  |  |  |
| MoBa | 48,024 | 75 |  |  |  |  |
| No | 12,474 (26.0) | 23 (30.7) | 12.6 (8.0, 19.0) | Ref | Ref | Ref |
| Yes | 35,461 (73.8) | 52 (69.3) | 8.8 (6.6, 11.5) | 0.57 (0.34, 0.93) | 0.56 (0.34, 0.94) | 0.56 (0.33, 0.93) |
| Missing | 89 (0.2) | 0 |  |  |  |  |
| Household crowding at birth (sqm/person) | |  |  |  |  |  |
| ABIS | 16,223 | 40 |  |  |  |  |
| >50 | 4482 (27.6) | 14 (35.0) | 14.1 (7.7, 23.6) | Ref | Ref | Ref |
| 25-50 | 9442 (58.2) | 17 (42.5) | 8.1 (4.7, 12.9) | 0.58 (0.28, 1.17) | 0.56 (0.27, 1.13) | 0.50 (0.20, 1.25) |
| <25 | 1131 (7.0) | 5 (12.5) | 19.9 (6.5, 46.4) | 1.42 (0.51, 3.94) | 1.02 (0.33, 3.19) | 1.58 (0.45, 5.58) |
| Missing | 1168 (7.2) | 4 (10.0) |  |  |  |  |
| MoBa | 62,764 | 91 |  |  |  |  |
| >50 | 31,588 (50.3) | 55 (60.4) | 10.8 (8.1, 14.1) | Ref | Ref | Ref |
| 25-50 | 25,997 (41.4) | 32 (35.2) | 7.7 (5.2, 10.8) | 0.71 (0.46, 1.10) | 0.74 (0.47, 1.15) | 0.75 (0.48, 1.18) |
| <25 | 2490 (4.0) | 3 (3.3) | 7.5 (1.6, 22.0) | 0.70 (0.22, 2.23) | 0.69 (0.21, 2.21) | 0.71 (0.22, 2.31) |
| Missing | 2689 (4.3) | 1 (1.1) |  |  |  |  |
| Early-life drinking water |  |  |  |  |  |  |
| ABIS | 10,863 | 29 |  |  |  |  |
| Public water | 8617 (79.3) | 21 (72.4) | 10.9 (6.8, 16.7) | Ref | Ref | Ref |
| Private water | 2200 (20.3) | 8 (27.6) | 16.3 (7.0, 32.1) | 1.49 (0.66, 3.37) | 1.39 (0.58, 3.30) | 1.74 (0.67, 4.52) |
| Missing | 46 (0.4) | 0 |  |  |  |  |
| MoBa | 62,764 | 91 |  |  |  |  |
| Public water | 56,732 (90.4) | 82 (90.1) | 9.0 (7.2, 11.2) | Ref | Ref | Ref |
| Private water | 5236 (8.3) | 8 (8.8) | 9.3 (4.0, 18.3) | 1.01 (0.49, 2.08) | 0.87 (0.40, 1.90) | 0.88 (0.41, 1.92) |
| Missing | 796 (1.3) | 1 (1.1) |  |  |  |  |
| Bed-sharing 0-6 months |  |  |  |  |  |  |
| MoBa | 75,229 | 108 |  |  |  |  |
| No | 57,909 (77.0) | 79 (73.1) | 8.5 (6.7, 10.6) | Ref | Ref | Ref |
| Yes | 16,680 (22.2) | 27 (26.0) | 10.1 (6.6, 14.6) | 1.18 (0.76, 1.83) | 1.21 (0.78, 1.88) | 1.22 (0.78, 1.90) |
| Missing | 650 (0.9) | 2 (1.9) |  |  |  |  |

^a^Adjusted model 1 (primary model): adjusted for sex, parental inflammatory bowel disease, parental origin, parental education level, maternal comorbidities (type 1 diabetes, autoimmune thyroid disease, rheumatoid arthritis). ^b^Adjusted model 2 (secondary model): also adjusted for delivery mode, maternal smoking, maternal age, birth weight, gestational age (weeks), full breastfeeding. ABIS, All Babies in Southeast Sweden study; CI, confidence interval; HR, hazard ratio; MoBa, The Norwegian Mother, Father and Child Cohort Study; PYR, person-year; Ref, reference category.

## **Supplementary Table 9.** Hygiene-related exposures of early-life and later risk of ulcerative colitis in the ABIS and MoBa cohorts

|  | **N** | **Events n** | **Incidence per 100,000 PYR** | **Unadjusted HR (95% CI)** | **Adjusted^a^ HR (95% CI)** | **Adjusted^b^**  **HR (95% CI)** |
| --- | --- | --- | --- | --- | --- | --- |
| Place of living at birth |  |  |  |  |  |  |
| ABIS | 16,223 | 57 |  |  |  |  |
| Urban | 11,923 (73.5) | 48 (84.2) | 18.1 (13.3, 24.0) | Ref | Ref | Ref |
| Rural | 3585 (22.1) | 8 (14.0) | 10.0 (4.3, 19.8) | 0.55 (0.26, 1.17) | 0.57 (0.27, 1.22) | 0.69 (0.23, 2.07) |
| Missing | 715 (4.4) | 1 (1.8) |  |  |  |  |
| MoBa | 101,270 | 95 |  |  |  |  |
| Urban | 95,221 (94.0) | 88 (92.6) | 5.6 (4.5, 6.9) | Ref | Ref | Ref |
| Rural | 4389 (4.3) | 5 (5.3) | 6.8 (2.2, 15.9) | 1.14 (0.46, 2.81) | 1.21 (0.49, 3.00) | 1.34 (0.54, 3.34) |
| Missing | 1660 (1.6) | 2 (2.1) |  |  |  |  |
| Having pets at birth |  |  |  |  |  |  |
| ABIS | 16,223 | 57 |  |  |  |  |
| No | 9210 (56.8) | 34 (59.6) | 16.6 (11.5, 23.2) | Ref | Ref | Ref |
| Yes | 6957 (42.9) | 23 (40.4) | 14.9 (9.4, 22.3) | 0.89 (0.53, 1.52) | 0.90 (0.52, 1.54) | 0.86 (0.36, 2.02) |
| Missing | 56 (0.8) | 0 |  |  |  |  |
| MoBa | 75,229 | 70 |  |  |  |  |
| No | 53,166 (70.7) | 47 (67.1) | 5.5 (4.1, 7.3) | Ref | Ref | Ref |
| Yes | 21,469 (28.5) | 22 (32.4) | 6.3 (4.0, 9.6) | 1.12 (0.67, 1.85) | 1.13 (0.67, 1.89) | 1.13 (0.67, 1.89) |
| Missing | 594 (0.8) | 1 (1.4) |  |  |  |  |
| Duration of having pets from birth to 36 mo | | |  |  |  |  |
| ABIS | 8667 | 33 |  |  |  |  |
| 0 year | 4127 (47.6) | 12 | 13.1 (6.8, 22.8) | Ref | Ref | Ref |
| 1 year | 1095 (12.6) | 5 | 20.5 (6.7, 47.8) | 1.57 (0.55, 4.46) | 1.78 (0.61, 5.13) | 1.10 (0.23, 5.38) |
| >2 years | 3424 (39.5) | 16 | 21.0 (12.0, 34.1) | 1.61 (0.76, 3.40) | 1.84 (0.84, 4.03) | 0.90 (0.28, 2.91) |
| Missing | 31 (0.4) | 0 |  |  |  |  |
| MoBa | 62,764 | 59 |  |  |  |  |
| 0 year | 39,027 (62.2) | 35 | 5.6 (3.9, 7.8) | Ref | Ref | Ref |
| 1 year | 8336 (13.3) | 10 | 7.3 (3.5, 13.4) | 1.20 (0.59, 2.41) | 1.19 (0.59, 2.43) | 1.19 (0.58, 2.43) |
| >2 years | 15,257 (24.3) | 14 | 5.7 (3.1, 9.5) | 0.96 (0.52, 1.79) | 0.95 (0.51, 1.79) | 0.96 (0.51, 1.81) |
| Missing | 144 (0.2) | 0 |  |  |  |  |
| Type of pet at birth |  |  |  |  |  |  |
| ABIS | 16,223 | 57 |  |  |  |  |
| No pets | 9210 (56.8) | 34 (59.6) | 16.6 (11.5, 23.2) | Ref | Ref | Ref |
| Cat | 2794 (17.2) | 10 (17.5) | 16.1 (7.7, 29.6) | 0.97 (0.48, 1.96) | 0.97 (0.47, 1.98) | 0.68 (0.19, 2.40) |
| Dog | 1821 (11.2) | 6 (10.5) | 14.8 (5.4, 32.3) | 0.89 (0.37, 2.12) | 0.89 (0.37, 2.14) | 1.07 (0.30, 3.78) |
| Other | 2342 (14.4) | 7 (12.3) | 13.4 (5.4, 27.7) | 0.81 (0.36, 1.82) | 0.81 (0.36, 1.85) | 0.91 (0.26, 3.22) |
| Missing | 56 (0.3) | 0 |  |  |  |  |
| MoBa | 75,229 | 70 |  |  |  |  |
| No pets | 53,166 (70.7) | 47 (67.1) | 5.5 (4.1, 7.3) | Ref | Ref | Ref |
| Cat | 9330 (12.4) | 10 (14.3) | 6.7 (3.2, 12.2) | 1.19 (0.60, 2.35) | 1.20 (0.60, 2.40) | 1.20 (0.60, 2.39) |
| Dog | 6681 (8.9) | 8 (11.4) | 7.4 (3.2, 14.6) | 1.32 (0.62, 2.79) | 1.32 (0.62, 2.81) | 1.34 (0.63, 2.85) |
| Other | 5458 (7.3) | 4 (5.7) | 4.5 (1.2, 11.5) | 0.76 (0.28, 2.12) | 0.78 (0.28, 2.17) | 0.76 (0.27, 2.14) |
| Missing | 594 (0.8) | 1 (1.4) |  |  |  |  |
| Older siblings at birth |  |  |  |  |  |  |
| ABIS | 16,223 | 55 |  |  |  |  |
| 0 (none) | 5941 (36.6) | 15 (26.3) | 11.3 (6.3, 18.7) | Ref | Ref | Ref |
| >1 | 9801 (60.4) | 40 (70.2) | 18.3 (13.1, 24.9) | 1.62 (0.89, 2.93) | 1.64 (0.91, 2.98) | 2.23 (0.81, 6.09) |
| Missing | 481 (3.0) | 2 (3.5) |  |  |  |  |
| MoBa | 101,270 | 95 |  |  |  |  |
| 0 (none) | 45,329 (44.9) | 44 (46.3) | 6.0 (4.3, 8.0) | Ref | Ref | Ref |
| >1 | 55,698 (55.1) | 51 (53.7) | 5.5 (4.1, 7.3) | 0.88 (0.59, 1.32) | 0.98 (0.64, 1.49) | 1.04 (0.63, 1.73) |
| Missing | 0 | 0 |  |  |  |  |
| Sibling order at birth |  |  |  |  |  |  |
| ABIS | 16,223 | 55 |  |  |  |  |
| 0 | 5941 (36.6) | 15 (27.3) | 11.3 (6.4, 18.7) | Ref | Ref | Ref |
| 1 | 5967 (36.8) | 23 (41.8) | 17.3 (11.0, 26.0) | 1.53 (0.80, 2.93) | 1.56 (0.81, 2.98) | 2.00 (0.69, 5.78) |
| 2 | 2612 (16.1) | 12 (21.8) | 20.7 (10.7, 36.1) | 1.82 (0.85, 3.90) | 1.84 (0.86, 3.94) | 2.87 (0.79, 10.40) |
| >3 | 1222 (7.5) | 5 (9.1) | 18.4 (6.0, 42.9) | 1.62 (0.59, 4.45) | 1.64 (0.59, 4.53) | 3.62 (0.61, 21.60) |
| Missing | 481 (3.0) | 0 |  |  |  |  |
| MoBa | 101,270 | 95 |  |  |  |  |
| 0 | 45,329 (44.8) | 44 (46.3) | 6.0 (4.3, 8.0) | Ref | Ref | Ref |
| 1 | 36,013 (35.6) | 29 (30.5) | 4.9 (3.3, 7.0) | 0.79 (0.50, 1.26) | 0.88 (0.54, 1.43) | 0.92 (0.53, 1.60) |
| 2 | 15,348 (15.2) | 18 (18.9) | 7.0 (4.2, 11.1) | 1.10 (0.63, 1.90) | 1.20 (0.68, 2.13) | 1.38 (0.71, 2.70) |
| >3 | 4337 (4.3) | 4 (4.2) | 5.5 (1.5, 14.1) | 0.85 (0.30, 2.36) | 0.99 (0.35, 2.79) | 1.26 (0.41, 3.87) |
| Missing | 243 (0.2) | 0 |  |  |  |  |
| Per additional older sibling | |  |  |  |  |  |
| ABIS | 15,742 | 55 | - | 1.22 (0.93, 1.61) | 1.23 (0.94, 1.61) | 1.57 (0.96, 2.59) |
| MoBa | 101,027 | 150 | - | 0.98 (0.78, 1.24) | 1.04 (0.82, 1.32) | 1.13 (0.84, 1.51) |
| Daycare by age 12 months |  |  |  |  |  |  |
| ABIS^c^ | 10,863 | 35 |  |  |  |  |
| No | 9136 (84.1) | 30 (85.7) | 14.8 (10.0, 21.1) | Ref | Ref | Ref |
| Yes | 625 (5.8) | 1 (2.9) | 7.2 (0.2, 39.9) | 0.48 (0.07, 3.54) | 0.48 (0.06, 3.51) | - |
| Missing | 1102 (10.1) | 4 (11.4) |  |  |  |  |
| MoBa | 62,764 | 59 |  |  |  |  |
| No | 56,861 (90.6) | 54 (91.5) | 5.9 (4.4, 7.7) | Ref | Ref | Ref |
| Yes | 5759 (9.2) | 5 (8.5) | 5.5 (1.8, 12.9) | 1.04 (0.42, 2.61) | 1.07 (0.43, 2.67) | 1.12 (0.45, 2.81) |
| Missing | 144 (0.2) | 0 |  |  |  |  |
| Daycare by age 36 months |  |  |  |  |  |  |
| ABIS | 8,677 | 33 |  |  |  |  |
| No | 893 (10.3) | 4 (12.1) | 20.1 (5.5, 51.5) | Ref | Ref | Ref |
| Yes | 7753 (89.4) | 29 (87.9) | 16.8 (11.3, 24.1) | 0.84 (0.29, 2.38) | 0.85 (0.30, 2.44) | 0.76 (0.17, 3.49) |
| Missing | 31 (0.4) | 0 |  |  |  |  |
| MoBa | 48,024 | 45 |  |  |  |  |
| No | 12,458 (25.9) | 7 (15.6) | 3.9 (1.6, 7.9) | Ref | Ref | Ref |
| Yes | 35,447 (73.8) | 38 (84.4) | 6.4 (4.6, 8.8) | 1.13 (0.50, 2.56) | 1.07 (0.47, 2.45) | 1.23 (0.51, 2.96) |
| Missing | 119 (0.2) | 0 |  |  |  |  |
| Household crowding at birth (sqm/person) | |  |  |  |  |  |
| ABIS | 16,223 | 57 |  |  |  |  |
| >50 | 4481 (27.6) | 13 (22.8) | 13.1 (7.0, 22.3) | Ref | Ref | Ref |
| 25-50 | 9462 (58.3) | 37 (64.9) | 17.6 (12.4, 24.2) | 1.34 (0.71, 2.53) | 1.31 (0.69, 2.46) | 1.28 (0.45, 3.62) |
| <25 | 1131 (7.0) | 5 (8.8) | 19.9 (6.5, 46.4) | 1.52 (0.54, 4.26) | 1.37 (0.48, 3.92) | 3.38 (0.82, 13.80) |
| Missing | 1149 (7.1) | 2 (3.5) |  |  |  |  |
| MoBa | 62,764 | 59 |  |  |  |  |
| >50 | 31,564 (50.3) | 31 (52.5) | 6.1 (4.1, 8.6) | Ref | Ref | Ref |
| 25-50 | 25,990 (41.4) | 25 (42.4) | 6.0 (3.9, 8.8) | 0.99 (0.58, 1.67) | 0.93 (0.55, 1.59) | 0.97 (0.57, 1.67) |
| <25 | 2488 (4.0) | 1 (1.7) | 2.5 (0.1, 14.0) | 0.42 (0.06, 3.08) | 0.41 (0.05, 2.99) | 0.43 (0.06, 3.16) |
| Missing | 2722 (4.3) | 2 (3.4) |  |  |  |  |
| Early-life drinking water |  |  |  |  |  |  |
| ABIS | 10,863 | 35 |  |  |  |  |
| Public water | 8623 (79.4) | 27 (77.1) | 14.1 (9.3, 20.5) | Ref | Ref | Ref |
| Private water | 2200 (20.3) | 8 (22.9) | 16.3 (7.1, 32.2) | 1.16 (0.53, 2.55) | 1.21 (0.54, 2.69) | 1.56 (0.59, 4.09) |
| Missing | 40 (0.4) | 0 |  |  |  |  |
| MoBa | 62,764 | 59 |  |  |  |  |
| Public water | 56,707 (90.3) | 57 (96.6) | 6.3 (4.7, 8.1) | Ref | Ref | Ref |
| Private water | 5,230 (8.3) | 2 (3.4) | 2.3 (0.3, 8.4) | 0.35 (0.08, 1.42) | 0.34 (0.08, 1.38) | 0.34 (0.08, 1.40) |
| Missing | 827 (1.3) | 0 |  |  |  |  |
| Bed-sharing 0-6 months |  |  |  |  |  |  |
| MoBa | 75,229 | 70 |  |  |  |  |
| No | 57,876 (76.9) | 46 (65.7) | 4.9 (3.6, 6.6) | Ref | Ref | Ref |
| Yes | 16,676 (22.2) | 23 (32.9) | 8.6 (5.4, 12.9) | 1.72 (1.05, 2.84) | 1.67 (1.01, 2.78) | 1.64 (0.98, 2.75) |
| Missing | 677 (0.9) | 1 (1.4) |  |  |  |  |

^a^Adjusted model 1 (primary model): adjusted for sex, parental inflammatory bowel disease, parental origin, parental education level, maternal comorbidities (type 1 diabetes, autoimmune thyroid disease, rheumatoid arthritis). ^b^Adjusted model 2 (secondary model): also adjusted for delivery mode, maternal smoking, maternal age, birth weight, gestational age (weeks), full breastfeeding. ABIS, All Babies in Southeast Sweden study; CI, confidence interval; HR, hazard ratio; MoBa, The Norwegian Mother, Father and Child Cohort Study; PYR, person-year; Ref, reference category.

## **Supplementary Table 10.** Pooled hazard ratios of inflammatory bowel disease by age <18 years

| **Characteristics** | **Unadjusted HR (95% CI)** | **Adjusted^a^ HR (95% CI)** | **Adjusted^b^**  **HR (95% CI)** |
| --- | --- | --- | --- |
| Place of living at birth |  |  |  |
| Urban | Ref | Ref | Ref |
| Rural | 1.17 (0.81, 1.70) | 1.14 (0.78, 1.67) | 1.35 (0.89, 2.07) |
| Having pets at birth |  |  |  |
| No | Ref | Ref | Ref |
| Yes | 1.10 (0.87, 1.42) | 1.08 (0.84, 1.39) | 1.09 (0.83, 1.43) |
| Duration of having pets from birth to 36 mo | | |  |
| 0 year | Ref | Ref | Ref |
| 1 year | 1.07 (0.72, 1.61) | 1.08 (0.74, 1.59) | 1.09 (0.74, 1.62) |
| >2 years | 1.05 (0.76, 1.46) | 1.01 (0.74, 1.39) | 0.95 (0.69, 1.33) |
| Type of pet at birth |  |  |  |
| No pet | Ref | Ref | Ref |
| Cat | 1.19 (0.86, 1.65) | 1.16 (0.83, 1.61) | 1.13 (0.76, 1.70) |
| Dog | 1.28 (0.89, 1.85) | 1.27 (0.88, 1.84) | 1.31 (0.85, 2.02) |
| Other | 0.85 (0.54, 1.35) | 0.78 (0.49, 1.26) | 0.84 (0.49, 1.45) |
| Older siblings at birth |  |  |  |
| 0 | Ref | Ref | Ref |
| >1 | 1.13 (0.92, 1.39) | 1.11 (0.81, 1.51) | 1.25 (0.97, 1.63) |
| Sibling order at birth |  |  |  |
| 0 | Ref | Ref | Ref |
| 1 | 1.06 (0.84, 1.34) | 1.10 (0.87, 1.40) | 1.20 (0.91, 1.59) |
| 2 | 1.21 (0.91, 1.61) | 1.20 (0.89, 1.62) | 1.35 (0.94, 1.94) |
| >3 | 1.34 (0.87, 2.06) | 1.36 (0.87, 2.12) | 1.66 0.96, 2.89) |
| Per additional older sibling |  |  |  |
|  | 1.10 (0.99, 1.23) | 1.11 (0.98, 1.24) | 1.21 (0.96, 1.52) |
| Daycare by age 12 months^c^ |  |  |  |
| No | Ref | Ref | Ref |
| Yes | 1.07 (0.32, 3.59) | 1.43 (0.93, 2.21) | - |
| Daycare by age 36 months | |  |  |
| No | Ref | Ref | Ref |
| Yes | 0.79 (0.44, 1.40) | 0.82 (0.41, 1.66) | 0.71 (0.49, 1.02) |
| Degree household crowding at birth (sqm/person) | | |  |
| >50 | Ref | Ref | Ref |
| 25-50 | 0.79 (0.60, 1.02) | 0.78 (0.60, 1.02) | 1.00 (0.38, 2.63) |
| <25 | 0.99 (0.55, 1.78) | 0.84 (0.46, 1.55) | 1.41 (0.47, 4.26) |
| Early-life drinking water |  |  |  |
| Public water | Ref | Ref | Ref |
| Private water | 1.11 (0.52, 2.36) | 1.01 (0.47, 2.15) | 1.13 (0.41, 3.14) |

^a^Adjusted model 1 (primary model): adjusted for sex, parental inflammatory bowel disease, parental origin, parental education level, maternal comorbidities (type 1 diabetes, autoimmune thyroid disease, rheumatoid arthritis). ^b^Adjusted model 2 (secondary model): also adjusted for delivery mode, maternal smoking, maternal age, birth weight, gestational age (weeks), full breastfeeding. ^c^Analyses did not converge because of too few events. HR, hazard ratio; CI, confidence interval; Ref, reference category. HR, hazard ratio; CI, confidence interval; Ref, reference category.

## **Supplementary Table 11.** STROBE Checklist for cohort studies

|  | Item No | Recommendation | Section |
| --- | --- | --- | --- |
| **Title and abstract** | 1 | (*a*) Indicate the study’s design with a commonly used term in the title or the abstract | Title, page 1 |
|  |  | (*b*) Provide in the abstract an informative and balanced summary of what was done and what was found | Abstract, page 2 |
| Introduction | | |  |
| Background/rationale | 2 | Explain the scientific background and rationale for the investigation being reported | Introduction, page 5 |
| Objectives | 3 | State specific objectives, including any prespecified hypotheses | Introduction, page 5, paragraph 3 |
| Methods | | |  |
| Study design | 4 | Present key elements of study design early in the paper | Methods, page 6-9 |
| Setting | 5 | Describe the setting, locations, and relevant dates, including periods of recruitment, exposure, follow-up, and data collection | Methods, page 6-8; “Study population”, “Main exposures”, “Outcomes” and Figure 1 |
| Participants | 6 | (*a*) Give the eligibility criteria, and the sources and methods of selection of participants. Describe methods of follow-up | Methods, page 6-8; “Study population”, “Main exposures”, “Outcomes”, “Data on covariates” and Figure 1 |
|  |  | (*b*) For matched studies, give matching criteria and number of exposed and unexposed | NA |
| Variables | 7 | Clearly define all outcomes, exposures, predictors, potential confounders, and effect modifiers. Give diagnostic criteria, if applicable | Methods, page 6-9; “Study population”, “Exposures”, “Outcome”, “Data on covariates” and Supplementary material; Supplementary Table 2 |
| Data sources/ measurement | 8* | For each variable of interest, give sources of data and details of methods of assessment (measurement). Describe comparability of assessment methods if there is more than one group | Methods, page 6-8 “Study population “Main exposures”, “Outcomes”, “Data on covariates”, Supplementary material; Supplemental Table 2 |
| Bias | 9 | Describe any efforts to address potential sources of bias | Methods, page 8; “Statistical analyses” and Discussion, page 15; “Strengths and limitations” |
| Study size | 10 | Explain how the study size was arrived at | Figure 1 |
| Quantitative variables | 11 | Explain how quantitative variables were handled in the analyses. If applicable, describe which groupings were chosen and why | Methods, page 7; “Outcomes”, page 8 “Data on covariates”, and “Statistical analysis” |
| Statistical methods | 12 | (*a*) Describe all statistical methods, including those used to control for confounding | Methods, page 8; “Statistical analysis” |
|  |  | (*b*) Describe any methods used to examine subgroups and interactions | Methods, page 8; “Statistical analysis” |
|  |  | (*c*) Explain how Missing were addressed | Methods, page 6-7; “Study population”, “Outcome”, and Figure 1, page 26 |
|  |  | (*d*) If applicable, explain how loss to follow-up was addressed | Figure 1, page 26 |
|  |  | (*e*) Describe any sensitivity analyses |  |
| Results | | |  |
| Participants | 13* | (a) Report numbers of individuals at each stage of study—eg numbers potentially eligible, examined for eligibility, confirmed eligible, included in the study, completing follow-up, and analyzed | Figure 1 |
|  |  | (b) Give reasons for non-participation at each stage | Figure 1 |
|  |  | (c) Consider use of a flow diagram | Figure 1 |
| Descriptive data | 14* | (a) Give characteristics of study participants (eg demographic, clinical, social) and information on exposures and potential confounders | Table 1, page 23 |
|  |  | (b) Indicate number of participants with Missing for each variable of interest | Supplementary Tables 7-9 |
|  |  | (c) Summarise follow-up time (eg, average and total amount) | Results, page 10 and Supplementary Table 4 |
| Outcome data | 15* | Report numbers of outcome events or summary measures over time | Results, page 10-12 and Figures 2-4 |
| Main results | 16 | (*a*) Give unadjusted estimates and, if applicable, confounder-adjusted estimates and their precision (eg, 95% confidence interval). Make clear which confounders were adjusted for and why they were included | Figures 2-4 |
|  |  | (*b*) Report category boundaries when continuous variables were categorized | Figures 2-4 |
|  |  | (*c*) If relevant, consider translating estimates of relative risk into absolute risk for a meaningful time period | NA |
| Other analyses | 17 | Report other analyses done—eg analyses of subgroups and interactions, and sensitivity analyses | Method, page 8; “Statistical analyses”, Results, page 11; “Pre-planned sub-analyses |
| Discussion | | |  |
| Key results | 18 | Summarize key results with reference to study objectives | Discussion, page 13, first paragraph |
| Limitations | 19 | Discuss limitations of the study, taking into account sources of potential bias or imprecision. Discuss both direction and magnitude of any potential bias | Discussion, page 15, “Strengths and limitations” |
| Interpretation | 20 | Give a cautious overall interpretation of results considering objectives, limitations, multiplicity of analyses, results from similar studies, and other relevant evidence | Discussion, page 13-16 |
| Generalizability | 21 | Discuss the generalizability (external validity) of the study results | Discussion, page 15, “Strengths and limitations” |
| Other information | | |  |
| Funding | 22 | Give the source of funding and the role of the funders for the present study and, if applicable, for the original study on which the present article is based | Page 17; “Funding” |

*Give information separately for exposed and unexposed groups.

# **Figures**


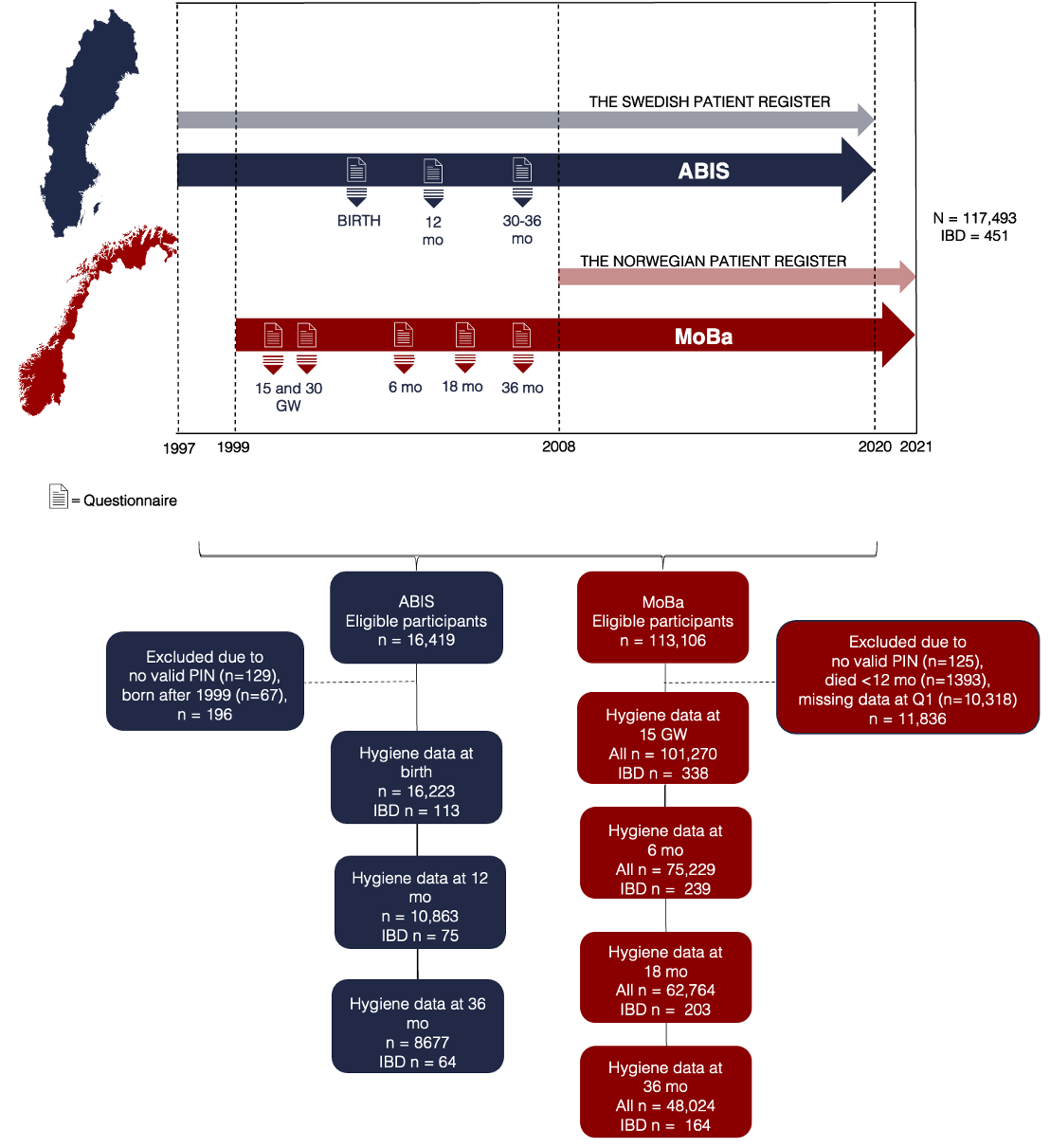


**Supplementary Figure 1.** Flowchart of study participants in the ABIS and MoBa cohorts. Inflammatory bowel disease was defined as at least two registrations of the International Classification of Diseases code for the disease. Analyses were performed using ABIS data retrieved at birth (place of living, pets, siblingship, household crowding), age 12 months (daycare, drinking water), and 36 months (daycare, pets). The MoBa data were retrieved at 15^th^ week of pregnancy (place of living, siblingship), age 6 months (bed-sharing, pets), 18 months (pets, daycare, household crowding, drinking water), and 36 months (daycare).


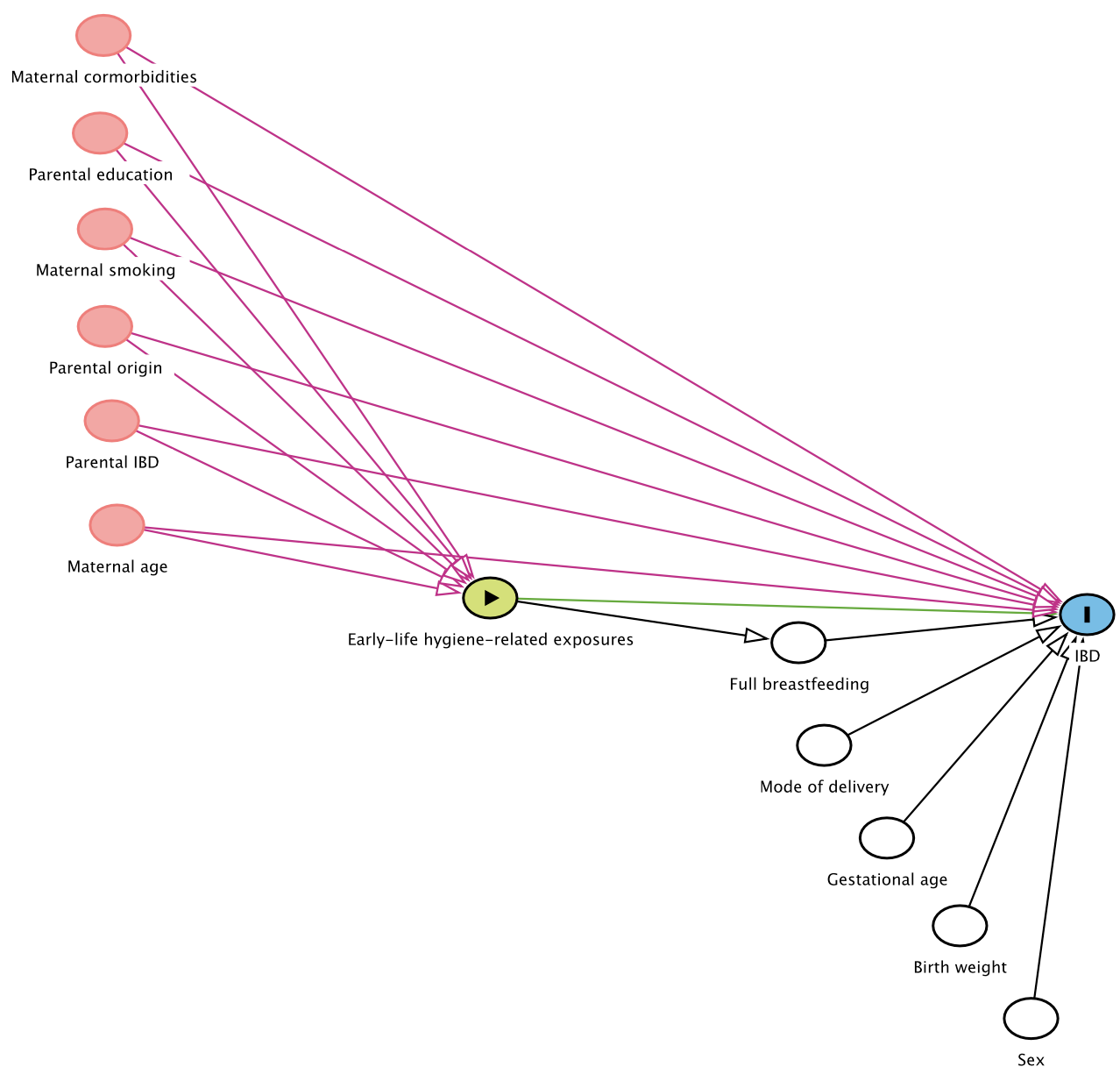


**Supplementary Figure 2.** Directed acyclic graph for hygiene-related exposures (green), the outcome inflammatory bowel disease (blue), confounding variables (red), and external variables only linked to IBD (white). We adjusted for all variables in our multivariate analyses (model 2).

# **References**

1. Agrawal M, Sabino J, Frias-Gomes C, et al. Early life exposures and the risk of inflammatory bowel disease: Systematic review and meta-analyses. *EClinicalMedicine*. Jun 2021;36:100884. doi:10.1016/j.eclinm.2021.100884

2. Amre DK, Lambrette P, Law L, et al. Investigating the hygiene hypothesis as a risk factor in pediatric onset Crohn's disease: a case-control study. *Am J Gastroenterol*. May 2006;101(5):1005-11. doi:10.1111/j.1572-0241.2006.00526.x

3. Baron S, Turck D, Leplat C, et al. Environmental risk factors in paediatric inflammatory bowel diseases: a population based case control study. *Gut*. Mar 2005;54(3):357-63. doi:10.1136/gut.2004.054353

4. Jakobsen C, Paerregaard A, Munkholm P, Wewer V. Environmental factors and risk of developing paediatric inflammatory bowel disease -- a population based study 2007-2009. *J Crohns Colitis*. Feb 2013;7(1):79-88. doi:10.1016/j.crohns.2012.05.024

5. Lee WS, Song ZL, Wong SY, et al. Environmental risk factors for inflammatory bowel disease: A case control study in Southeast Asian children. *J Paediatr Child Health*. May 2022;58(5):782-790. doi:10.1111/jpc.15830

6. Radon K, Windstetter D, Poluda AL, Mueller B, von Mutius E, Koletzko S. Contact with farm animals in early life and juvenile inflammatory bowel disease: a case-control study. *Pediatrics*. Aug 2007;120(2):354-61. doi:10.1542/peds.2006-3624

7. Strisciuglio C, Giugliano F, Martinelli M, et al. Impact of Environmental and Familial Factors in a Cohort of Pediatric Patients With Inflammatory Bowel Disease. *J Pediatr Gastroenterol Nutr*. Apr 2017;64(4):569-574. doi:10.1097/mpg.0000000000001297

8. Ball TM, Castro-Rodriguez JA, Griffith KA, Holberg CJ, Martinez FD, Wright AL. Siblings, day-care attendance, and the risk of asthma and wheezing during childhood. *N Engl J Med*. Aug 24 2000;343(8):538-43. doi:10.1056/nejm200008243430803

9. Nicolaou NC, Simpson A, Lowe LA, Murray CS, Woodcock A, Custovic A. Day-care attendance, position in sibship, and early childhood wheezing: a population-based birth cohort study. *J Allergy Clin Immunol*. Sep 2008;122(3):500-6.e5. doi:10.1016/j.jaci.2008.06.033

10. Rantala AK, Magnus MC, Karlstad Ø, et al. Is the Association of Early Day Care Attendance with Childhood Asthma Explained by Underlying Susceptibility? *Epidemiology*. May 2020;31(3):451-458. doi:10.1097/ede.0000000000001163

11. Irgens LM. The Medical Birth Registry of Norway. Epidemiological research and surveillance throughout 30 years. *Acta Obstet Gynecol Scand*. Jun 2000;79(6):435-9.

12. Statistics Norway. One in ten live in a crowded dwelling. [<https://www.ssb.no/en/bygg-bolig-og-eiendom/artikler-og-publikasjoner/one-in-ten-live-in-a-crowded-dwelling>] Published 13 September, 2017. Accessed 3 October, 2022.

13. Everhov AH, Halfvarson J, Myrelid P, et al. Incidence and Treatment of Patients Diagnosed With Inflammatory Bowel Diseases at 60 Years or Older in Sweden. *Gastroenterology*. Feb 2018;154(3):518-528 e15. doi:10.1053/j.gastro.2017.10.034
